# hiPSC-Derived Cardiomyocyte Model of LQT2 Syndrome Derived from Asymptomatic and Symptomatic Mutation Carriers Reproduces Clinical Differences in Aggregates but Not in Single Cells

**DOI:** 10.3390/cells9051153

**Published:** 2020-05-07

**Authors:** Disheet Shah, Chandra Prajapati, Kirsi Penttinen, Reeja Maria Cherian, Jussi T. Koivumäki, Anna Alexanova, Jari Hyttinen, Katriina Aalto-Setälä

**Affiliations:** 1 Faculty of Medicine and Health Technology and BioMediTech Institute, Tampere University, 33520 Tampere, Finland; chandra.prajapati@tuni.fi (C.P.); kirsi.penttinen@tuni.fi (K.P.); reeja.maria.cherian@tuni.fi (R.M.C.); jussi.koivumaki@tuni.fi (J.T.K.); anna.alexanova@tuni.fi (A.A.); jari.hyttinen@tuni.fi (J.H.); katriina.aalto-setala@tuni.fi (K.A.-S.); 2 Heart Hospital, Tampere University Hospital, 33520 Tampere, Finland

**Keywords:** *HERG*, LQT2, channelopathies, in vitro electrophysiology, arrhythmia, induced pluripotent stem cells, in-silico modeling

## Abstract

Mutations in the *HERG* gene encoding the potassium ion channel HERG, represent one of the most frequent causes of long QT syndrome type-2 (LQT2). The same genetic mutation frequently presents different clinical phenotypes in the family. Our study aimed to model LQT2 and study functional differences between the mutation carriers of variable clinical phenotypes. We derived human-induced pluripotent stem cell-derived cardiomyocytes (hiPSC-CM) from asymptomatic and symptomatic *HERG* mutation carriers from the same family. When comparing asymptomatic and symptomatic single LQT2 hiPSC-CMs, results from allelic imbalance, potassium current density, and arrhythmicity on adrenaline exposure were similar, but a difference in Ca^2+^ transients was observed. The major differences were, however, observed at aggregate level with increased susceptibility to arrhythmias on exposure to adrenaline or potassium channel blockers on CM aggregates derived from the symptomatic individual. The effect of this mutation was modeled in-silico which indicated the reactivation of an inward calcium current as one of the main causes of arrhythmia. Our in-vitro hiPSC-CM model recapitulated major phenotype characteristics observed in LQT2 mutation carriers and strong phenotype differences between LQT2 asymptomatic vs. symptomatic were revealed at CM-aggregate level.

## 1. Introduction

Genetic long QT syndrome (LQTS) is an autosomal dominant disorder with a prevalence of 1 in 2000 people in the Caucasian population and its prevalence has been estimated to be 1 in 250–500 in Finland [1,2,3]. LQTS type2 (LQT2) is the second most common cause of LQTS with symptoms of syncope, seizures and sudden cardiac death due to ventricular arrhythmia [4,5,6,7,8]. LQT2 is a channelopathy with a mutation in the *HERG* gene on chromosome 7 [9,10]. *HERG* or *KCNH2* codes for the α-subunits of one of the principle cardiac repolarizing potassium (K^+^) channel current i.e., the rapid component of delayed-rectifier repolarizing potassium current (IKr). A mutation here typically reduces IKr and increases the repolarization time which could lead to arrhythmias [4]. Arrhythmias in LQT2 may be triggered by physical or emotional stress, adrenergic rush during acoustic stimuli, fright, or occur during rest or sleep [11].

More than 300 mutations have been found in the *HERG* gene that could induce LQT2 [11,12]. Individuals with mutations in *HERG* present a varying clinical phenotype depending on factors including type, topology and the location of the mutation in the *HERG* gene, gender, single nucleotide polymorphisms (SNP), modifier genes, co-morbidities and age. [4,11,13,14]. Missense mutations, pore segment mutations, mutations in the α-helix of the channel and female gender increase the risk for arrhythmias [3,4,15,16,17,18,19]. Some mutation carriers present symptoms while some continue to remain asymptomatic with or without a prolonged QT interval. These individuals may still have an increased risk for QT prolongation, could develop symptoms later in life and are at a higher risk of sudden death compared to non-carriers [20,21]. Asymptomatic carriers of LQT2 are recommended life-style modifications and beta-blocker therapy, especially patients with abnormal T-wave morphology in the electrocardiogram (ECG) in women and the pore location of mutation in men [16,22]. There is a clear unmet medical need to study the mechanism of clinically silent LQT2, especially in countries like Finland with a higher prevalence of LQTS.

Animal models and mammalian expression systems have limitations in replicating human pathophysiology. Animal models also have ethical concerns, high costs and there is a need for models to replicate human physiology and genetic variation [23]. Human-induced pluripotent stem cell-derived cardiomyocytes (hiPSC-CMs) have been shown to recapitulate disease phenotypes of inherited cardiac diseases such as long QT syndrome and catecholamine polymorphic ventricular tachycardia (CPVT) [24,25,26,27,28,29]. However, previously published models of LQT2 have not compared the asymptomatic and symptomatic phenotypes in-vitro from the same mutation apart from a study by Chai et al., which implicated that modifier genes were responsible for phenotypic dissimilarity [14].

The p.L552S mutation from our study was a missense mutation located on the transmembrane α-helix of segment S5 forming the pore of the α-subunits of the HERG channel [15,30]. Our general aim was to investigate whether hiPSC-CMs could recapitulate the phenotype differences of clinically symptomatic and asymptomatic mutation carriers. To elucidate the disease mechanisms, we studied (1) the localization of the wild type and mutated ion channel, (2) ion channel function and calcium dynamics (3) arrhythmia propensity of cardiomyocyte (CM) aggregates by micro electrode array (MEA) under potassium ion channel block and adrenaline exposure. Our results highlighted that hiPSC-CM models can reveal phenotypical differences of mutation carriers with different clinical findings.

## 2. Materials and Methods

### 2.1. Patient Characteristics

Skin biopsies from two healthy controls and two individuals carrying the p.L552S mutation in *HERG* were used for this study. Healthy control cells were derived from a 55-year-old female (UTA.04602.WT) and from a 34-year-old male (UTA.04511.WT). One of the mutation carriers (1) is an asymptomatic 19-year-old male and two lines from him were used in this study (UTA.03412.LQT2A and UTA.03417.LQT2A). The other mutation carrier (2) is a symptomatic 44-year-old female and two lines from her were used in this study (UTA.03809.LQT2A and UTA.03810.LQT2A). A signed informed consent was obtained from all individuals who participated in this study. This study was approved by the Ethics Committee of Pirkanmaa Hospital District to establish, culture and differentiate hiPSC lines (R08070).

### 2.2. Genetic Screening

Next-generation sequencing (NGS) consisting of a panel of 254 genes, including the assessment of non-coding variants, was studied with the ’Comprehensive Cardiology Panel Plus Analysis´ at Blueprint Genetics, Helsinki, Finland (https://blueprintgenetics.com). cDNA was extracted from primary fibroblasts of the mutation carrier**s** using the Machery–Nagel NucleoSpin Tissue XS kit, and was delivered to the company for this service. The panel covered known genetic causes of channelopathies and cardiomyopathies and included a sequence analysis and copy number variation analysis of the following genes: AARS2, ABCC6*, ABCC9, ACAD9, ACADVL, ACTA1, ACTA2, ACTC1, ACTN2, AGK*, AGL, AGPAT2, AKAP9, ALMS1*, ALPK3, ANK2, ANO5, APOA1, ATPAF2, BAG3, BRAF*, CACNA1C*, CACNB2, CALM1*, CALM2, CALM3, CALR3, CAPN3, CASQ2, CASZ1, CAV3, CBL, CDH2, CHRM2, COX15, CPT2, CRYAB, CSRP3, CTNNA3, DBH, DES, DMD, DNAJC19, DOLK, DPM3, DSC2, DSG2, DSP, DTNA, DYSF, EEF1A2, ELAC2, EMD, ENPP1, EPG5, ETFA, ETFB, ETFDH, FAH, FBXL4, FBXO32, FHL1*, FHOD3, FKRP, FKTN, FLNC*, FOXD4*, FOXRED1, FXN*, GAA, GATA4*, GATA5, GATA6, GATAD1, GATC*, GBE1, GFM1, GLA, GLB1, GMPPB, GSK3B, GTPBP3, GUSB*, HADHA, HAND1, HAND2, HCN4, HFE, HRAS, IDUA, ILK, ISPD, JPH2, JUP, KCNA5, KCNE1, KCNE2, KCNH2, KCNJ2, KCNJ5, KCNQ1, KLHL24, KRAS*, LAMA2, LAMP2, LARGE, LDB3, LEMD2, LMNA, LMOD2, LRRC10, LZTR1, MAP2K1, MAP2K2, MAP3K8, MIPEP*, MLYCD, MRPL3*, MRPL44, MRPS22, MT-ATP6, MT-ATP8, MT-CO1, MT-CO2, MT-CO3, MT-CYB, MT-ND1, MT-ND2, MT-ND3, MT-ND4, MT-ND4L, MT-ND5, MT-ND6, MT-RNR1, MT-RNR2, MT-TA, MT-TC, MT-TD, MT-TE, MT-TF, MT-TG, MT-TH, MT-TI, MT-TK, MT-TL1, MT-TL2, MT-TM, MT-TN, MT-TP, MT-TQ, MT-TR, MT-TS1, MT-TS2, MT-TT, MT-TV, MT-TW, MT-TY, MTO1#, MYBPC3, 118 MYBPHL, MYH6, MYH7, MYL2, MYL3, MYL4, MYO18B, MYOT, MYPN, MYRF, NDUFAF2, NEXN, NF1*, NKX2–5, NONO, NOS1AP, NRAP, NRAS, NUP155, PARS2, PCCA, PCCB#, PKP2*,#, PLEC, PLEKHM2, PLN, PNPLA2, POMT1, PPA2, PPCS, PPP1CB, PRDM16, PRKAG2, PTPN11, QRSL1, RAF1, RASA2, RBCK1, RBM20, RIT1, RMND1*, RRAS, RYR2, SALL4, SCN10A, SCN1B, SCN3B, SCN5A, SCNN1B, SCNN1G, SCO1, SCO2, SDHA*, SELENON#, SGCA, SGCB, SGCD, SGCG, SHOC2, SLC22A5, SLC25A20, SLC25A3, SLC25A4, SMCHD1, SOS1, SOS2, SPEG, SPRED1, STAG2, TAB2, TAZ, TBX20*, TBX5, TCAP, TECRL, TGFB3, TMEM43, TMEM70, TNNC1, TNNI3, TNNI3K, TNNT2, TOR1AIP1, TPM1, TRDN, TRIM32, TRPM4, TSFM#, TTN*, TTR, VARS2, VCL, VCP, VPS13A and *XK*.

The target region for each gene included the coding exons and ±20 base pairs from the exon-intron boundary. Genes with suboptimal coverage in the assay were marked with (#) and genes with partial or whole gene segmental duplications in the human genome were marked with an asterisk (*) if they overlapped with the UCSC pseudogene regions (https://blueprintgenetics.com/tests/panels/cardiology/comprehensive-cardiology-panel/).

### 2.3. hiPSC Generation, Culture and Characterization

The derivation of one control line (UTA.04602.WT) was characterized previously [28,31]. The control cell line UTA.04511.WT was generated by Sendai viral transduction, and LQT2 cell lines UTA.03412.LQT2A, UTA.03417.LQT2A, UTA.03809.LQT2A and UTA.03810.LQT2A were generated by retroviral transduction, with all the lines characterized similarly to the control line UTA.04602.WT.

### 2.4. Cardiomyocyte Differentiation

Human-induced pluripotent stem cells (hiPSCs) were differentiated into CMs by the mouse endoderm-like cell line END-2 co-culture method [32] on mouse embryonic fibroblast (MEF) feeder cells (CellSystems, Troisdorf, Germany). Cardiac aggregates were spontaneously beating clusters of the cardiac hiPSC-CMs manifesting in the hiPSC differentiation plate. The CMs were left to mature for at least 30 days before they were used in experiments and maintained in KnockOut-Dulbecco’s modified eagle’s media (KO-DMEM) supplemented with 20% fetal bovine serum (FBS). The CMs used in experiments were typically between 30–60 days post induction of differentiation for consistency. These aggregates were dissected and used directly on MEA experiments or dissociated into single cells and used for voltage clamp and Ca^2+^ imaging experiments.

### 2.5. Allelic Imbalance

Total RNA from beating cardiomyocyte aggregates was extracted with Total RNA Purification Plus kit (Norgen Biotek Corp, Thorold, ON, Canada) and reverse transcribed with High Capacity cDNA Reverse Transcription kit (Applied Biosystems, Carlsbad, CA, USA). The expression ratios of HERG Finnish founder mutation (FinA) (p.L552S mutation) and HERG-wild type (WT) alleles were determined by plasmid-derived standard curve method (Moretti et al. 2010). Plasmids carrying HERG-FinA and WT alleles were produced as described in the supplementary section (Figure S8). The standard curve was made by including 1:0, 8:1, 4:1, 2:1, 1:1, 1:2, 1:4, 1:8 and 0:1 (WT:MUT) plasmid mixes into the reaction plate. Each mix contained in total 1 ng/μL of plasmid DNA. Quantitative polymerase chain reaction (qPCR) was performed simultaneously for UTA.03809.LQT2A, UTA.03810.LQT2A, UTA.03412.LQT2A and the plasmid standards with a Custom Taqman SNP Genotyping Assay (ID AH20Z2J, Life Technologies Ltd., see the supplementary section Figure S8 for primer sequences). All samples and standards were analyzed in triplicate. The reactions were performed with Applied Biosystems 7300 Real-Time PCR system (Applied Biosystems, Foster City, CA, USA). Thermal conditions of the amplification step were 2 min at 50 °C, 10 min at 95 °C, 40 cycles of 15 s at 95 °C and 1 min at 60 °C. Background fluorescence and fluorescence after amplification were measured from each well with a pre-read run and a post run respectively using default settings. The expression ratios of the two HERG alleles for the samples were estimated from the standard curve based on their average ΔCt (Ct mut−Ct wt) values as described in the supplementary section (Figure S8).

### 2.6. HERG Channel Expression in HEK-293 Expression System

The expression vector for the wild type (WT) HERG expression was constructed by subcloning the HERG cDNA from pSP64-HERG1a (Addgene plasmid # 53051, a gift from Michael Sanguinetti) in the C-terminal of the mScarlet fluorescent protein cDNA at the BamHI (Thermo Fisher Scientific # FD0054) and HindIII (Thermo Fisher Scientific # FD0504) sites of pmScarlet-i_C1 (Addgene plasmid # 85044, kindly provided by Dorus Gadella). The p.L552S mutation was introduced into the HERG_mScarlet fusion plasmid construct by QuikChange site directed-mutagenesis technique, using the forward primer- gccgtgctgttctcgctcatgtgcaccttt and reverse primer- aaaggtgcacatgagcgagaacagcacggc. The mutation was confirmed by direct sequencing using a primer specific for vector sequence which flanked the HERG cDNA. For transfection, the HEK-293 cells were maintained in Dulbecco’s modified eagle’s media (DMEM, GIBCO) supplemented with 10% Fetal Bovine Serum (FBS, GIBCO), 1% penicillin/streptomycin (Sigma) and Glutamax in 5% CO_2_ at 37 °C. The cells were seeded at a density of 2 × 10^5^ onto coverslips in a Nunclon 12-well plate. Transfection was carried out according to the protocol for FuGene^®®^ lipofection reagent and five hours post-transfection the cell media was replaced with serum-free starvation media. Twelve hours post-transfection, the cells were fixed in 4% paraformaldehyde in preparation for confocal microscopy and the coverslips were removed and mounted onto glass microscope slides using Vectashield’s antifade mounting medium with a fluorescent DNA stain (DAPI). Cells were then stored at 4 °C overnight before being examined under Zeiss LSM 780 Laser Scanning Confocal Microscope.

### 2.7. Ca^2+^ Imaging

Ca^2+^ imaging experiments were performed as described previously [33]. In addition to baseline studies, the effect of 10 nM adrenaline (Sigma) was studied. For the Ca^2+^ analysis, regions of interest were selected for spontaneously beating cells and background noise was subtracted before further data processing. In addition, the Ca^2+^ transients (CaT) were analyzed by identifying the signals as normal or abnormal and categorizing the abnormalities into subgroups according to their abnormality types. Data of the WT CMs (UTA.04602.WT and UTA.04511.WT), asymptomatic CMs (UTA.03412.LQT2A and UTA.03417.LQT2A) and symptomatic CMs (UTA.03809.LQT2A and UTA.03810.LQT2A) were pooled due to similar Ca^2+^ peak parameter values. Ca^2+^ data was analyzed in a ClampFit 10.5 software and statistical analysis of Ca^2+^ data was made with SPSS software version 24 (SPSS, Chicago, IL, USA). 

### 2.8. Voltage Clamp

The hiPSC-CMs were perfused with an extracellular solution containing 143 mM NaCl, 4.8 mM KCl, 1.8 mM CaCl_2_, 1.2 mM MgCl_2_, 5 mM glucose and 10 mM 4-(2-hydroxyethyl)-1-piperazineethanesulfonic acid (HEPES) (pH was adjusted to 7.4 with NaOH). The intracellular solution contained 132 mM KMeSO_4_, 20 mM KCl, 1 mM MgCl_2_, 4 mM ethylene glycol-bis(β-aminoethyl ether)-N,N,N′,N′-tetraacetic acid (EGTA) and 1 mM CaCl_2_ (pH was adjusted to 7.2 with KOH). The extracellular solution was heated to 35 °C–36 °C with an inline heater SH-27B, controlled with a TC-324B controller unit (Warner Instruments Inc., Hamden, CT, USA). The patch electrodes had a tip resistance of 2.0–4.0 MΩ with intracellular solution. Perforated patch using Amphotericin B was performed to record currents in Axon Series 200B patch-clamp amplifier connected to a Digidata 1440a AD/DA converter driven by pCLAMP 10.2 software (all from Molecular Devices LLC, San Jose, USA). In the voltage-clamp protocol, the holding potential (HP) was set to −50 mV with a voltage step from −40 mV to 40 mV of 4 s and a step size of 20 mV. In addition, 5 µM nimodipine and 10 µM chromanol 293B were added into the extracellular solution to block the L-type Ca^2+^ current and slow delayed rectifier potassium current (IKs) respectively. The IKr current was isolated by subtracting the currents before and after the addition of 1 µM E-4031. The peak and tail currents were calculated from the end of the test pulse and the peak of the tail current, respectively. Ionic currents were divided by cell capacitances and presented as pA/pF.

### 2.9. Micro Electrode Array (MEA) Electrophysiology

The MEA recording and analysis was performed as described in [33]. The effect of IKr and IKs block was studied by using E4031 (Sigma) and JNJ303 (Tocris), respectively, at 300 nM. Vehicular control used in these experiments was the solvent used to dissolve the drug, in this case, distilled water for E4031 and DMSO for JNJ303. Four different concentrations of adrenaline (Sigma-Aldrich, St. Louis, MO, USA) (100 nM, 300 nM, 600 nM and 1 µM) were assessed. Vehicle control of distilled water was used. The method of measuring the field potential duration (FPD) was done until the end of the repolarization peak reaching the abscissa at 0 µV. This was done on a basis that the peak of the field potential (FP) repolarization was more closely related to 50% of the action potential duration [34] instead of the whole repolarization time. The data was analyzed using Origin 2017 (OriginLab, Northampton, MA, USA). The FP signal analysis was semi-automated using an in-house written module in Origin, that could compute the beat rate (BPM), inter-beat intervals (IBIs) and field potential durations (FPDs). Due to variability in signal morphology, the FPD end point detection was done by two ways, automated as well as user defined semi-automated, for accurate end point detection. To reduce variability in the analysis, the signals from the baseline and the condition were analyzed consecutively. Bazett’s formula was used to calculate the beat-rate-corrected FPD (cFPD). The aggregates chosen for the study were normalized to reduce the impact of the beating rate. Aggregates with a beating frequency below 20 or above 90 BPM were not chosen for the study to prevent over or under-correction. 

### 2.10. Mathematical Modeling and Computer Simulations of CM Function

To elucidate the arrhythmogenic mechanisms of the LQT2 mutation, we employed a previously published mathematical model of the electrophysiology and ion dynamics in hiPSC-CM (ref to [35]). The in-silico hiPSC-CM model was updated to improve robustness by modifying the conductance values of If (hyperpolarization activated repolarizing funny current), IKr (delayed rapid repolarizing K^+^ current), bCa (inward background Ca^2+^ current), bNa (inward background Na^+^ current), NCX (Na^+^-Ca^2+^ exchanger current), NKA (Na^+^-K^+^ ATPase current) and PMCA (outward plasma membrane Ca^2+^ current) to 0.0281, 0.0363, 0.0029, 0.00095, 2636, 13.1 and 0.06, respectively. The previous model version, which was parameterized based on averaging a large collection of in vitro data, was very sensitive to perturbations in IKr, as shown in the supplementary Figure S6 in the original publication [34]. The new parameter set was used to increase the robustness of repolarization, so that simulations could be run more consistently to compare the in-silico and in-vitro effects of LQT and drugs. To simulate the effect of IKr block by E4031 and IKs block by JNJ303 in parallel in a healthy and mutated virtual CM, we implemented the latter variant by reducing the conductance of IKr by 30%, in line with in-vitro findings of this study. Simulation results were recorded from pacing experiments at 1 Hz frequency. The MATLAB implementation of the hiPSC-CM model will be available at ResearchGate networking site and on request from the authors.

### 2.11. Statistics

The data from the two healthy controls, LQT2 asymptomatic and symptomatic hiPSC-CMs were pooled respectively and the standard error of the mean (SEM) between them was shown by error bars in the voltage clamp, calcium imaging and microelectrode array experiments. Comparison within the cell lines (before and after drug administration) was performed with non-parametric Wilcoxon test. Between the healthy controls, LQT2 asymptomatic and symptomatic subsets comparison was done with a non-parametric Kruskal–Wallis test with Bonferroni correction for the calcium imaging experiments and the Kruskal–Wallis test with Dunn’s multiple comparison test for voltage clamp and MEA data. *p* < 0.05 (*), *p* < 0.01 (**), *p* < 0.001 (***) were considered statistically significant. Data has been expressed as average ± SEM where n refers to the number of CMs or the number of aggregates.

## 3. Results

A 44-year-old female patient was diagnosed with LQTS with a QT/QTc of 492/470 ms. She had experienced episodes of arrhythmia, syncope, asthma and presented hearing impairment. She showed characteristics of LQTS patients with periods of non-sustained ventricular tachycardia (NSVT) on 24 h Holter recording. β-blocker treatment was initially prescribed and a cardiac defibrillator (ICD) was implanted later for prevention of potentially fatal arrhythmia. Genetic screening of her family members confirmed a gene mutation in p.L552S responsible for LQTS type-2 in her and in her 19-year-old-son. The son had juvenile rheumatoid arthritis, with methotrexate and occasional corticosteroid treatments. Typically, his QTc was in the normal range (QT/QTc of 450/398, Figure S9), but once in the 24-h Holter recording it was reported to be up to 500 ms. No arrhythmias were reported by this mutation carrier and none were present in any clinical recordings. To understand the variability in this disease phenotype, hiPSC-CMs were generated from the dermal fibroblasts from these two mutation carriers and two healthy volunteers to determine if this genotype–phenotype difference could be observed invitro.

The fibroblasts were reprogrammed by retroviral transfection (except UTA.04511.WT which was reprogrammed by Sendai viral transduction) with pluripotency genes *Sox2*, *Nanog*, *Oct3/4* and *c-Myc*. Multiple clones were generated, cultured, expanded and stored. All the hiPSC lines showed typical hiPSC colony morphology (Figure S1) and qPCR confirmed the expression of endogenous pluripotency markers and silenced exogenous markers (Figure S2). The qPCR of RNA of embryoid bodies confirmed the expression of endodermal, mesodermal and ectodermal genes (Figure S3). A normal karyotype was confirmed in all the lines (Figure S4). Immunofluorescence imaging confirmed the presence of pluripotency proteins (Figure S5). Cardiac differentiation was carried out by the END-2 method using MEF feeder cells, and beating cardiac cells were over 30 days of age when they were further characterized and studied. 

**Figure 1:**
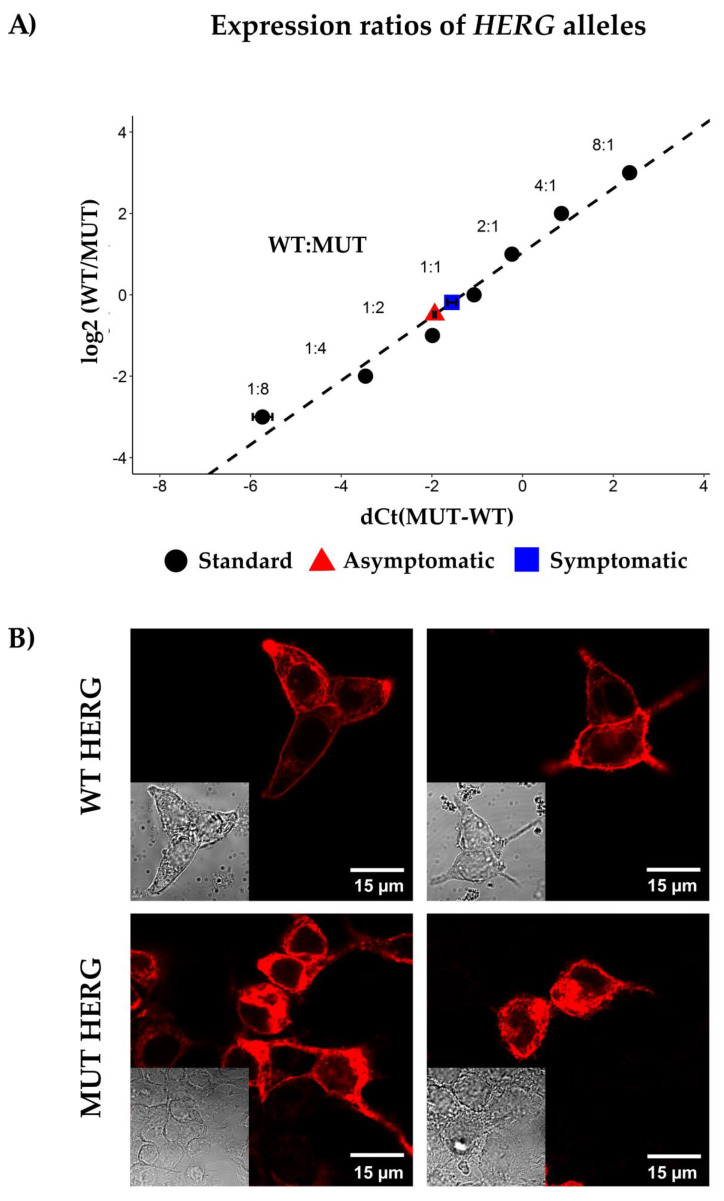
Expression ratios of the wild type (WT) and the mutant (MUT) *HERG* alleles and localization of the HERG protein. (**A**) Allelic imbalance determination with the standard curve method. Standard data points were plotted as log2 (WT plasmid/MUT plasmid) ratios vs. corresponding mean ΔCt values (Ct(MUT)–Ct(WT)). Standard curve (dashed line) was determined with the least squares fit method. Expression ratios of the *HERG* alleles in the long QT syndrome type-2 (LQT2) samples were calculated using the mean ΔCt values of the samples and the standard curve equation. Mean ΔCt value for the LQT2 asymptomatic was calculated from the triplicates of the UTA.03412.LQT2 human-induced pluripotent stem cell-derived cardiomyocyte (hiPSC-CM) samples and the mean ΔCt value for the symptomatic was calculated from the UTA.03809.LQT2 and the UTA.03810.LQT2 hiPSC-CM samples. Error bars indicate the standard errors of ΔCt means. The locations of the data points indicate that the *HERG* (*KCNH2)* alleles are expressed in all three cell lines with the wild type/mutant allele ratio somewhere between 1:2 and 1:1. The allelic imbalance assay was repeated with another set of samples of the three cell lines, yielding similar results (data not shown). (**B**) The fluorescent mScarlet-tagged WT and MUT *HERG* alleles expressed in the HEK293 cells indicated the increased localization to the cell membrane in the wildtype (WT) and the increased cytoplasmic localization of mutant HERG (MUT *HERG*) (transmitted light images added as inset).

To elucidate whether an allelic imbalance between the symptomatic and asymptomatic LQT2 mutation carriers was responsible for the phenotype discord, we conducted an allelic discrimination study. The wild-type to mutant allele ratio of *HERG* expression was between 1:2 and 1:1 in samples from both asymptomatic and symptomatic LQT2 mutation carriers (Figure 1A). The ratio 1:1 signifies an equal expression of both alleles and the ratio 1:2 signifies that there is twice as much mRNA of the mutant allele than the wild type allele in the sample. The mutant allele was expressed more than the wild type allele on the mRNA level in samples from both LQT2 mutant hiPSC-CMs. This experiment showed similar levels of allelic expression in the asymptomatic and the symptomatic LQT2 samples (Figure 1A), thus ruling out the difference in mutant and the wild type allele expression to affect the clinical phenotype. Genetic screening by NGS analysis of a panel of 254 genes by the ´Comprehensive cardiology panel´ from Blueprint Genetics identified only a heterozygous missense variant *KCNH2* c.1655T>C, p.Leu552Ser (p.L552S) in both of the LQT2 mutation carriers. No other pathogenic, likely pathogenic or variants of uncertain significance (VUS) were observed in this panel in either individual. This screening panel also covered ~2000 non-coding disease-causing variants. This suggests that there were no other pathogenic genetic variants in the genes known to affect the heart phenotype in the mutation carriers that could explain the difference in their clinical phenotype.

To understand the molecular and cellular effects of the p.L552S mutation, we expressed the WT and the mutant *HERG* mScarlet fusion plasmid construct in HEK293 cells which did not express endogenous HERG. The localization of the exogenously expressed WT and mutant HERG was determined by confocal microscopy. The mutated HERG expressed in HEK293 exhibited an altered localization pattern with respect to the WT HERG (Figure 1B). HEK293 cells expressing WT protein showed an increased expression of HERG at the cell membrane with much lower levels of expression in the cytoplasm whereas the mutant protein was more expressed in the cytoplasmic compartment. This suggested that the p.L552S mutation may have had a detrimental effect on the cellular processing of the HERG protein that could lead to the trafficking deficiency (Figure 1B).

**Figure 2:**
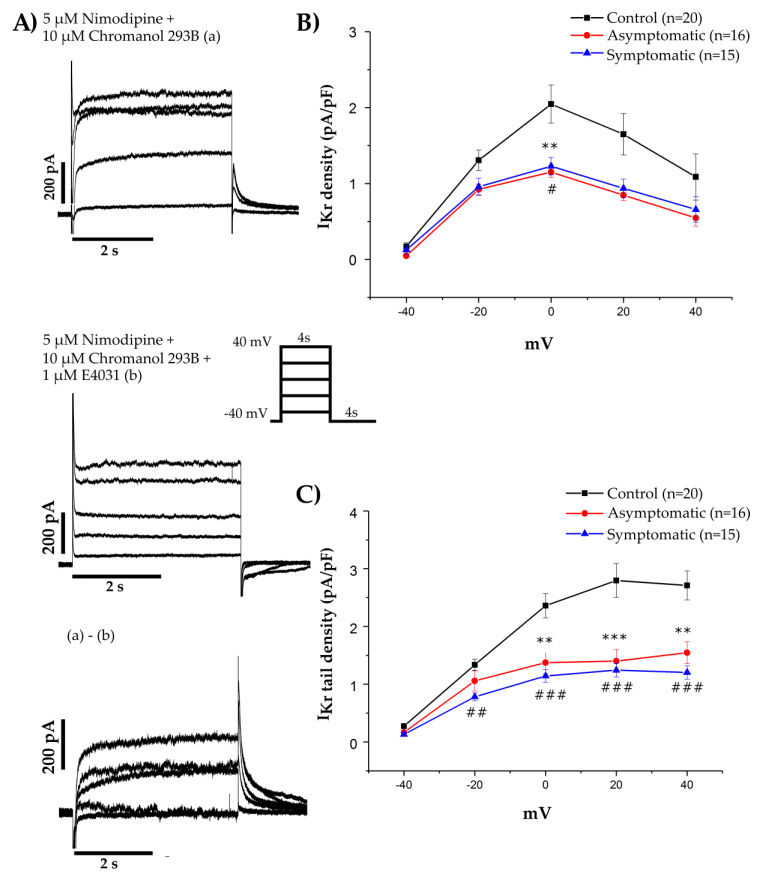
IKr densities: (**A**) IKr currents were obtained as 1 µM E-4031 sensitive currents with the used protocol. Inset: the voltage protocol used to elicit the IKr current. (**B**,**C**) Current-voltage plot of the peak IK_r_ current densities and the tail IK_r_ densities respectively from controls (black), for the LQT2 asymptomatic (red), and symptomatic (blue) hiPSC-CMs. Values inside parentheses indicate the number of hiPSC-CMs analyzed. Data is presented as the mean ± SEM, (*) *p* < 0.05, (**) *p* < 0.01 and (***) *p* < 0.001 between control and asymptomatic and (#) *p* < 0.05, (##) *p* < 0.01 and (###) *p* < 0.001 between control and symptomatic hiPSC-CMs.

We next analyzed the functionality of the HERG channel by studying the electrophysiological properties at single-cell level by patch-clamp technique (Figure 2). The delayed-rectifier repolarizing potassium current (IKr) affected due to the mutation; was studied using voltage-clamp experiments, which revealed that the IKr peak and tail current densities in the hiPSC-CMs from both the asymptomatic and the symptomatic LQT2 mutation carriers were significantly lower compared to the hiPSC-CMs from the healthy controls (Figure 2A–C, *p* < 0.05, ANOVA). No significant difference was found in the IKr current densities between the asymptomatic and symptomatic LQT2 hiPSC-CMs. 

**Figure 3:**
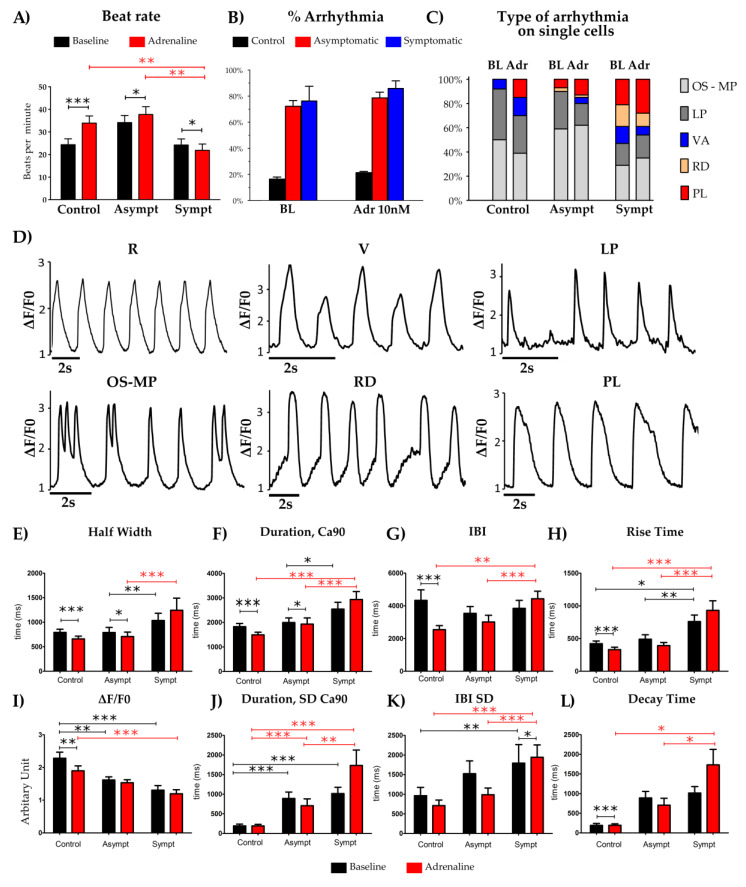
LQT2 hiPSC-CM shows increased calcium transient abnormalities. (**A**) Response of the control, the LQT2 asymptomatic and the symptomatic hiPSC-CMs to application of 10 nM adrenaline. (**B**) Percentage of CMs showing arrhythmia at baseline (BL) and during 10 nM adrenaline (Adr 10nM) perfusion. (**C**) Comparison of type of arrhythmia observed at baseline and under adrenaline (Adr) in the control, the LQT2 asymptomatic and the symptomatic CMs. (**D**) Representative arrhythmia observed in the control, the LQT2 asymptomatic and the symptomatic hiPSC-CMs Ca^2+^ transients. LQT2 asymptomatic CMs have most occurrences of oscillations and low amplitude peaks, while symptomatic CMs have quite equal amounts of all the five kinds of abnormalities but more delay in rise and decay phase than the LQT2 asymptomatic. RG = Regular, OS = oscillations and multiple peaks, LP = low amplitude peaks, PL = plateau abnormality/delay in decay, RD = rise delay, VA = varying amplitude. (**E**–**L**) Ca^2+^ imaging parameters at baseline and in the presence of 10 nM adrenaline. Data is presented for n = 68, 78, 44 for the control, the LQT2 asymptomatic, and the symptomatic respectively, as the mean ± SEM, (*) *p* < 0.05, (**) *p* < 0.01 and (***) *p* < 0.001.

Single dissociated hiPSC-CMs were further analyzed with Ca^2+^ imaging at baseline and under the effect of adrenaline. A representative CaT is shown in Figure S6A. The application of adrenaline significantly increased the mean beat rate (BPM) in the control (*p* < 0.001) and the LQT2 asymptomatic (*p*= 0.042) but significantly reduced the beating rate in the symptomatic line (*p* = 0.037) (Figure 3A). Both LQT2-CMs derived from symptomatic or asymptomatic presented with increased arrhythmias at baseline and in the presence of adrenaline (Figure 3B). The different type of CaT abnormalities (Figure 3C,D) were categorized into the following groups as also described previously [36,37]: oscillation (OS), if Ca^2+^ oscillated for two or more peaks without reaching the baseline, low amplitude peaks (LP), which had small amplitude Ca^2+^ events of at least 10% of the preceding Ca^2+^ spike amplitude, plateau abnormality (PL), if the decay time of the Ca^2+^ was prolonged, rise delay (RD), if the rise time of the Ca^2+^ was prolonged and varying amplitude (VA), if the amplitude of the peaks were varying continuously in the trace. CaT was categorized as regular (RG), if the trace did not include any of the aforementioned abnormalities. The symptomatic hiPSC-CMs presented an increased percentage of RD (rise delay) arrhythmia and PL (plateau abnormality) arrhythmias compared to both the healthy controls and the LQT2 asymptomatic hiPSC-CMs (Figure 3C). At baseline compared to the hiPSC-CMs from the LQT2 asymptomatic mutation carrier, the hiPSC-CMs from the symptomatic patient had increased the CaT half-width (Figure 3E) (*p* = 0.003), increased the CaT duration, Ca90 (Figure 3F) (*p* = 0.025) and increased the rise time (Figure 3H) (*p* = 0.002). The hiPSC-CMs from the symptomatic patient had a reduced BPM and an increased inter-beat interval (IBI) after adrenaline application (Figure 3G) and increased standard deviation in the Ca90 and IBI (Figure 3J,K). The basal CaT characteristics of LQT2-CMs showed significantly reduced Ca^2+^ levels calculated by ∆F/F0 compared to the controls (*p* <0.005), Figure 3I). ANOVA comparison between the three lines showed that the rise time and the decay time of the CaT from the symptomatic lines were found to be significantly higher after adrenaline application compared to the other lines (*p* < 0.001 and *p* < 0.05, respectively) (Figure 3H,L).

**Figure 4:**
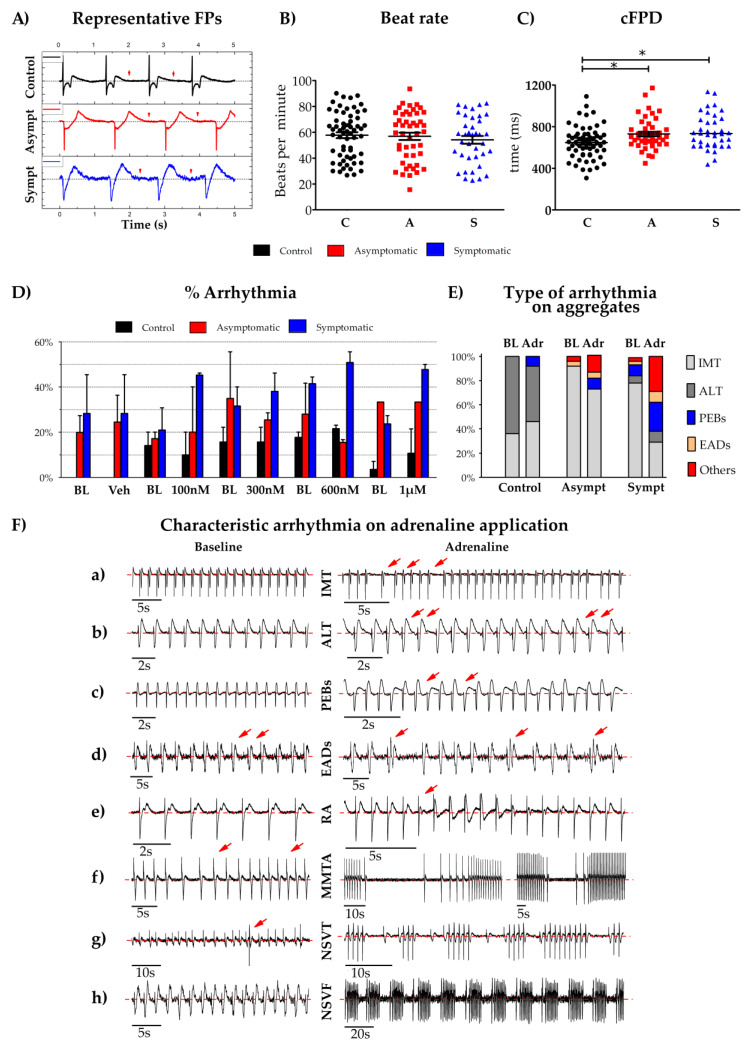
LQT2 hiPSC-CMs from the symptomatic patients presenting with increased arrhythmia on adrenaline application (**A**) Representative field potential traces from the control, the LQT2 asymptomatic and the symptomatic aggregates at baseline. The red arrows depict the end point of the repolarization duration, i.e., the field potential duration (FPD); the FPD was calculated from the start of the depolarization wave until the repolarization wave reaching the base. (**B**,**C**) The scatter-dot plots show the beat rate and the corrected field potential durations (cFPDs) from the control, the LQT2 asymptomatic and the symptomatic (labelled as (C,A and S with black, red and blue colors respectively), the boxes show the mean and SEM, for n = 62, 46, 38, respectively, for both the plots. (**D**) Percentage of aggregates showing arrhythmia at baseline, vehicle and under 100 nM, 300 nM, 600 nM and 1 µM adrenaline application; n numbers for the control, the LQT2 asymptomatic and the symptomatic; Bl-Veh n = 20, 19, 20, Bl–Adr100nM n = 22, 17, 22, Bl-Adr300nM n= 20, 16, 23, BL-Adr600nM n = 23, 19, 22 and BL-Adr1µM n = 24, 18, 21, respectively. (**E**) Comparison of the types of arrhythmia observed at baseline and under adrenaline (types elaborated below). (**F**) Representative field potentials and characteristic arrhythmia observed in the control, the LQT2 asymptomatic and the symptomatic hiPSC-CM aggregates at baseline and after the application of adrenaline. (**a**) IMT = irregular monomorphic tachycardiac-like arrhythmia and variation of field potential beats, (**b**) ALT = alternans, (**c**) PEBs = premature ectopic beats, (**d**) EADs = early after polarization-like arrhythmia, (**e**) RA = Repolarization abnormality with monomorphic arrhythmia (**f**) MMTA = Monomorphic triggered activity-like arrhythmia (**g**) NSVT = Non-sustained ventricular tachycardia-like arrhythmia, (**h**) NSVF = Non-sustained ventricular fibrillation-like arrhythmia. The red arrows point to distinguish the types of arrhythmia for clarity purposes.

Then, we studied the electrophysiological properties of hiPSC-CM aggregates by using micro electrode array (MEA). Representative field potential signals are presented in Figure 4A. The MEA analysis revealed that though the control and LQT2 aggregates showed a similar beating rate, the corrected field potential duration (cFPD) of the LQT2 aggregates was significantly increased compared to the controls (Figure 4B,C). The baseline characteristics did not show differences between the LQT2 asymptomatic and the symptomatic hiPSC-CM aggregates. An instantaneous rise in adrenaline in the body is a known cause of major arrhythmia like ventricular tachycardia and torsades de pointes (TdP) in LQT2 patients, so we exposed CM aggregates to different concentrations of adrenaline. All three cell lines showed a linear increase in the beating rate and a decrease in the field potential duration with increasing adrenaline concentration (Figure S7A–C). Adrenaline induced the occurrence of arrhythmias, also often observed clinically. The arrhythmias were defined on the basis of their rate of occurrence, the beating frequency, change in morphology compared to regular baseline beats and the length of time for which they occur. The different arrhythmias that were observed were: irregular monomorphic tachycardia (IMT), alternans (ALT) with alternating long and short inter-beat intervals, premature ectopic beats (PEBs), early after depolarization-like arrhythmias (EADs), repolarization abnormalities with monomorphic arrhythmia (RA), monomorphic triggered activity (MMTA), non-sustained ventricular tachycardia-like arrhythmia (NSVT) and non-sustained ventricular fibrillation (NSVF)-like arrhythmia, all found more frequently in hiPSC-CMs aggregates from the symptomatic mutation carriers (Figure 4D–F). MMTA was defined as a triggered activity lasting ≥10 s without any change in the morphology of the FP waveform and without premature beats as a trigger. NSVT was defined as a triggered activity requiring a premature ectopic beat as a trigger, with activity lasting ≥30 s. NSVF was defined as a triggered activity with an increased beating rate, requiring a premature ectopic beat as a trigger, with >6 consecutive ventricular complexes or ventricular tachycardia -type VT lasting <200 s.

**Figure 5:**
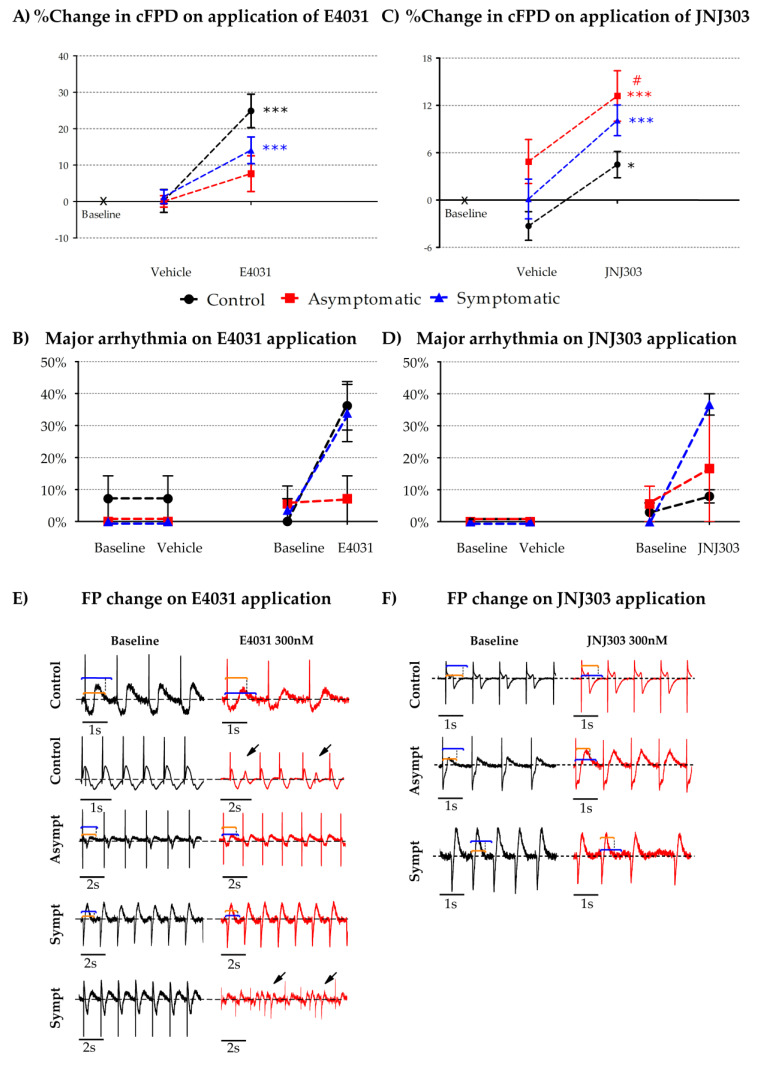
The effects of the IKr and IKs blockers (**A**,**B**) The plots show the responses of the control, the LQT2 asymptomatic and the symptomatic hiPSC-CM aggregates to the IKr blocker E4031 at 300 nM and the occurrence of major arrhythmia for the vehicle (distilled H_2_O) n = 26, 18, 19 respectively, and for 300 nM E4031 n = 39, 17, 24, respectively. (**C**,**D**) Responses of the control, the LQT2 asymptomatic and the symptomatic hiPSC-CM aggregates to the IKs blocker JNJ303 300 nM and the occurrence of major arrhythmia for the vehicle (DMSO) n = 9, 3, 11, respectively, and 300 nM JNJ303 n = 27, 13, 12, respectively. Data is presented as mean ± SEM, (*) *p* < 0.05 and (***) *p* < 0.001 compared between Long QT asymptomatic or symptomatic and control and (#) *p* <0.05, compared on all three lines by ANOVA. (**E**,**F**) Representative field potentials and the characteristic arrhythmia observed in the control, the LQT2 asymptomatic and the symptomatic hiPSC-CM aggregates at baseline (black) and after application of E4031 and JNJ303 (red); the prolongation in FPD is marked by orange and blue lines, whereas EAD-like arrhythmias and premature ectopic beats (PEBs) are marked with black arrowheads.

To understand if there were differences in the repolarization reserve between the LQT2 asymptomatic and the symptomatic mutation carrier-derived hiPSC-CM aggregates, we tested two drugs that are known to prolong the QT duration. We applied both IKr specific blocker E4031 and IKs specific blocker JNJ303 each at concentrations of 300 nM. Interestingly, we found that E4031 significantly prolonged the corrected field potential duration (cFPD) in the control (*p* < 0.0001) and the symptomatic (*p* = 0.001) but not in the LQT2 asymptomatic hiPSC-CM aggregates (paired-t test at baseline and after 300 nM E4031 (Figure 5A). The early after polarization-like arrhythmia (EAD)s, premature ectopic beats (PEB)s and the ceasing of beating (labeled as major arrhythmia) were more commonly observed in the symptomatic compared to the LQT2 asymptomatic aggregates (Figure 5B). On the application of the IKs blocker JNJ303, the controls (*p* = 0.03), the LQT2 asymptomatic (*p* = 0.0002) and the symptomatic (*p* = 0.0008), all were observed to have a significantly prolonged cFPD with the LQT2 aggregates being more affected compared to the controls (Figure 5C). The asymptomatic line also showed a more significant percentage increase in cFPD (13.19%) in comparison to the controls (4.49%) and the symptomatic (10.10%), determined by ANOVA (*p* = 0.01) (Figure 5C). The IKs block by JNJ303 also evoked EAD-like arrhythmias, PEBs and ceasing of beating more commonly in the symptomatic compared to the LQT2 asymptomatic and the healthy control aggregates (Figure 5D). Representative field potentials and characteristic arrhythmias in the control, the LQT2 asymptomatic and the symptomatic hiPSC-CM aggregates are presented in Figure 5E,F. No statistically significant differences were observed when just the vehicle was applied. Testing the probability of arrhythmia occurrence by the two tailed Fisher´s exact test and odds ratio between the arrhythmia occurrences between the symptomatic and the asymptomatic hiPSC-CMs, for concentrations of adrenaline 100 nM, 300 nM, 600 nM and 1 µM, 300nM E4031 and 300nM JNJ303, the odds ratios of each of the tests were 2.7, 1.8, 5.33, 1.9, 2.3 and 3.6, respectively. This indicated a higher probability of the symptomatic being more arrhythmic compared to the asymptomatic hiPSC-CMs. The *p* values were found to be significantly different only for 600 nM (*p* = 0.04).

**Figure 6:**
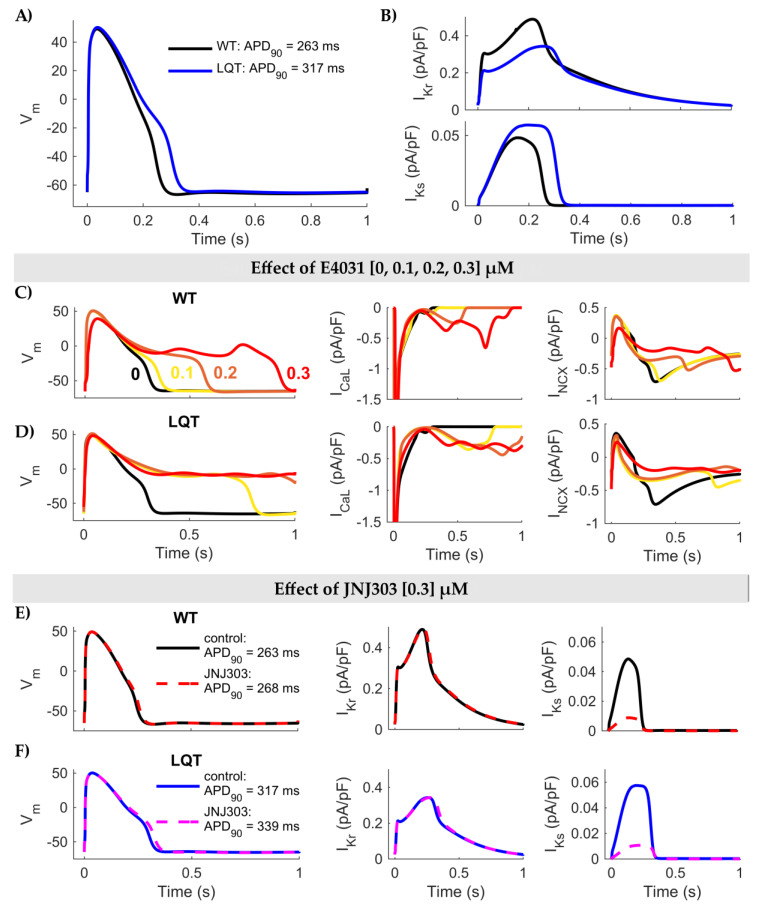
Drug responses of healthy controls vs. LQT2 hiPSC-CMs in-silico. (**A**) Comparison of AP from the healthy control hiPSC (black) and the LQT2 (blue) showing a prolongation of 20.53% in APD90. (**B**) Effect of p.L552S *HERG* mutation on the densities of K^+^ currents, with a reduction in IKr, but an increase in IKs. (**C**,**D**) Effect of the IKr blocker E4031 at 0 µM (black), 0.1 µM (yellow), 0.2 µM (orange) and 0.3 µM (red) on AP morphology (left panel), in healthy control hiPSC and LQT2, and the effect of the IKr block 0%–100% on the L-type calcium channel (middle panel) and the sodium channel exchanger NCX current (Sodium calcium exchanger- calcium efflux current) (right). (**E**,**F**) Effect of the IKs blocker JNJ303 on the control (top) and the LQT2 (bottom) with baseline as (solid line) 0.3 µM JNJ303 (dotted line) on the AP morphology. Finally, the effect of the IKs block 0%–100% on the densities of IKr and IKs, shown using black solid and red dotted lines for the controls and blue solid and pink dotted lines for the LQT2, respectively.

We employed computational modeling to elucidate the mechanisms underlying arrhythmogenic events in LQT2. Figure 6A,B show the predicted differences in the action potential (AP) morphology and ion currents of interest: IKr and IKs. The increased action potential duration (APD) was in line with the cFPD prolongation data we obtained in-vitro on MEA. Interestingly, there was a small compensatory increase in IKs in the LQT2 vs. the healthy controls, due to the delayed repolarization in LQT2 (Figure 6B). The simulation of the effects of the varying E4031 concentrations illustrated the increased inducibility of repolarization abnormalities in LQT2 (Figure 6C,D), in line with a reduced repolarization reserve. Furthermore, the in-silico results suggested that the reactivation of ICaL (L- type calcium current) was one of the mechanisms for repolarization failure, in the middle panel of Figure 6C,D. As expected, based on a smaller contribution to repolarization, the impact of the IKs blocker JNJ303 was rather mild in the simulations. As shown in Figure 6E,F, the IKs blocker increased the APD by 6.9% in LQT2 and 1.9% in the controls, because both the absolute and the relative contribution of the IKs was larger in LQT2.

## 4. Discussion

Clinically, around 25% of patients of all LQTS-carrying genetic mutations have a normal QT interval, and they do not present a disease phenotype, although they might still retain an increased risk of having cardiac events [20]. This difference in clinical phenotype has challenged the detection and treatment of these LQT2 asymptomatic mutation carriers, and studies suggest that their prevalence could be higher as they often go undetected [1]. Both the symptomatic and the asymptomatic mutation carriers from LQT2 have been reported to have an increased risk of getting cardiac events or sudden death [3,4,15,16,17,18,19]. In this study, we focused on investigating the potential cellular differences using hiPSC technology with cells derived from asymptomatic LQT2 and symptomatic LQT2. 

### 4.1. What Is the Mechanism for Reduced HERG Channel Function?

It has previously been reported for some autosomal dominant mutations that allelic imbalance could lead to a variation in the phenotype for identical genotypes. The increased allelic imbalance in the BRCA1 gene is associated with increased breast cancer risk [38] and SNPs (single nucleotide polymorphsims) on the mutated allele of KCNQ1, causing allelic imbalance leading to a less severe form of LQT1. These findings partially explain the reason for variable disease penetrance in breast cancer and LQT1 respectively [39]. In our study, allelic imbalance showed that the mutant and WT alleles were expressed equally in both the LQT2 asymptomatic and the symptomatic hiPSC-CMs between 1:1 and 1:2 ratios of WT:MUT (Figure 1A). This indicated that there could be other dominating factors involved in phenotype difference.

The HERG channel, being a membrane protein, is difficult to crystallize and currently no crystal structure exists in the Protein Data Bank (PDB) and only recently studies on homology models or Cryogenic electron microscopy (cryo-EM) structures of the HERG structure have been published [40,41]. Therefore, it has been difficult to understand or predict the effect of the mutation on the structure and function. Wild type mature HERG protein is a 155 kDa glycosylated protein and shows up as two bands on the Western blot at 155 kDa and 135 kDa, containing both mature trafficked and immature protein, which was retained in the endoplasmic reticulum (ER) [15]. The p.L552S mutation in our carriers is a pore domain missense mutation that lay on the α-helix of the S5 transmembrane segment, forming the pore of the α-subunits of the HERG channel [15,30]. A study by Anderson et al. (2014) reported that the mutation p.L552S could be a trafficking defect by Western blot, though the confocal images of the cellular and sub-cellular localization of the channel were not reported in the study [15]. To confirm the localization of the mutated p.L552S mutation, we transiently expressed the mutation in HEK293 cells and saw a reduced membrane expression, corroborating previous findings that p.L552S is a protein trafficking defect mutation (Figure 1B).

In our study, we found that both the peak and tail IKr current density mediated by the HERG channel to be significantly reduced in all LQT2 hiPSC-CMs compared to the controls. These data combined with the 1:2–1:1 WT:MUT allelic expression ratios and the reduced membrane localization of the ion channel showed the p.L552S mutation has an aberrant channel trafficking. No difference was observed between the hiPSC-CMs from the LQT2 asymptomatic and the symptomatic mutation carriers, indicating that the HERG channel function was equally impaired in both and other factors contributed towards the phenotype differences. This finding is in concurrence with a previously published LQT2 hiPSC-CM model with symptomatic and asymptomatic forms of p.R752W mutation which reported no significant differences in the IKr density [14].

### 4.2. What Is Causing Arrhythmogenic Events in LQT2?

Ca^2+^ imaging of the single hiPSC-CMs showed significantly increased standard deviation in the Ca^2+^ transient (CaT) durations and inter-beat intervals. The increased arrhythmia in both the asymptomatic and the symptomatic LQT2 hiPSC-CMs could be attributed to this increased beat rate variation as also reported in past studies [42,43]. Interestingly, in our study, β-adrenergic stimulation did not lead to an increase in the beating frequency of hiPSC-CMs from the symptomatic patients (Figure 3). Adrenaline exposure can develop calcium cycling abnormalities and delayed after depolarizations (DADs) which occasionally suppress the following action potential, thus preventing the increase in beating frequency. Adrenaline caused more PL and OS arrhythmias in symptomatic CMs which increased the duration of one abnormal peak and therefore decreased the beat rate [44]. This phenomenon of a blunted β-adrenergic response at single hiPSC-CMs has been reported in the pathogenic variants of mutations in titin mutations of dilated cardiomyopathy [45,46].

The significantly slower rise and decay time after application of adrenaline in the symptomatic hiPSC-CMs suggested that there were alterations in the calcium release and uptake, which could be investigated in future studies. In our study β-adrenergic stimulation evoked arrhythmia in both the asymptomatic and the symptomatic hiPSC-CMs. Previous studies on hiPSC-CMs from LQT1 asymptomatic mutation carriers have reported that β-adrenergic stimulation evoked arrhythmia at the single-cell level [25], whereas single hiPSC-CMs generated from LQT2 asymptomatic mutation carriers did not evoke arrhythmia [47]. Our study is the first to analyze cardiac phenotypes in CM aggregates and in single CMs, as well as to report that the clinical differences in phenotypes can be observed in aggregates which may not be seen in single CMs. In the future it is important to consider this aspect e.g., when conducting pre-clinical drug toxicity tests.

Clinically, the disease phenotype of LQT2 and the onset of characteristic TdP arrhythmia or VT-like arrhythmia have been reported to be due to sudden arousal, exercise, sudden emotional or acoustic triggers or at rest during sleep, and are preceded by a pause in R–R interval with a transient dramatic rise in the QT interval immediately before the TdP [7,11,48]. T-wave alternans, T-wave liability (beat-to-beat variation) or oscillation in the ECG have been reported to be a precursor to lethal arrhythmia and major cardiac events in humans and in rabbit LQT2 models [49,50]. On clinical examination our symptomatic patient developed non-sustained ventricular tachycardia on a 24 h Holter recording while on beta-blocker bisoprolol and was implanted with an implantable cardioverter-defibrillator (ICD). In our in-vitro studies, we successfully reproduced the adrenaline-induced arrhythmia including irregular monomorphic tachycardiac-like arrhythmia, alternans (ALT), EAD-like arrhythmias, PEBs, repolarization abnormality with monomorphic triggered activity, NSVT and NSVF-like arrhythmia (Figure 4F), more commonly in the hiPSC-CM aggregates from the symptomatic patient compared to the LQT2 asymptomatic and the healthy controls (Figure 4D,E). These findings suggested that the distinction in phenotype was more pronounced at the CM-aggregate level compared to the single-cell where both the LQT2 asymptomatic and symptomatic derived hiPSC-CMs were similarly arrhythmic. Multiple hypotheses have been proposed for the underlying mechanisms of TdP, clinically including the prolongation of action potential duration and the multi foci of triggered activity [51]. Another hypothesis for TdP is the reduced dispersion of repolarization (DOR) across the heart wall, where some CMs repolarize more rapidly than others with prolonged repolarization [51,52]. A study on a canine LQT2 model reported pharmacologically enhanced connexin 43 gap junction coupling, prevented arrhythmias by reducing the DOR [53]. This phenomenon could explain why the hiPSC-CM aggregates from the p.L552S mutation presented with the clinical phenotype more prominently than the single dissociated hiPSC-CMs in our study. To our knowledge, this phenomenon has not been reported in cellular levels before. Clinically, the increased risk of arrhythmias for females has been reported [3,4,15,16,17,18,19] but in in-vitro experiments the effect of the gender from whom the hiPSC-CMs are derived, and its effect on CM-function remains to be investigated.

The IKr density was the same in the symptomatic and the asymptomatic LQT2 hiPSC-CMs, but there was still a difference in the arrhythmia propensity in the aggregates. This suggested the presence of compensatory outward currents rescuing the phenotype in the LQT2 asymptomatic hiPSC-CMs, or inward currents increasing arrhythmia in the symptomatic, or both. The compensatory outward current would be increasing the repolarization reserve; a parameter reflecting the capacity of CMs to repolarize and a risk factor for arrhythmia when reduced [54]. A recently published report on LQT2 implicated two modifier genes to provide an explanation for phenotype differences; causing increased L-type calcium currents in symptomatic and a compensatory outward K^+^ current in LQT2 asymptomatic hiPSC-CMs [14].

To explore if the hiPSC-CMs from the symptomatic mutation carriers were more susceptible to the reduction in the repolarization reserve, we tested the effect of the compounds E4031 and JNJ303 which reduced the capacity of repolarization by blocking IKr and IKs, respectively. Interestingly, the IKr block prolonged the cFPD in hiPSC-CMs from the LQT2 symptomatic but not in asymptomatic (Figure 5). A past study of LQT1 symptomatic and asymptomatic hiPSC-CMs on MEA did not report any major differences in the effects of the application of IKs blockers [55]. In the current study, IKs block prolonged the cFPD more than the control but similarly for the symptomatic and the asymptomatic LQT2 hiPSC-CM aggregates (Figure 5). Both IKr and IKs block evoked EAD-like arrhythmia, PEBs and the ceasing of beating, more commonly in the symptomatic compared to the LQT2 asymptomatic or healthy controls (Figure 5). Our findings suggest compensatory outward currents in the LQT2 asymptomatic, which did not exist in the symptomatic, rescue the repolarization reserve.

The results from the mathematical modeling suggested that the arrhythmia-causing mechanism in the LQT2 hiPSC-CMs (EAD generation and repolarization failure) was the reactivation of L-type calcium currents (Figure 6). This could explain the prolonged cFPD and arrhythmias in the hiPSC-CMs from the symptomatic patient. It could be speculated that the increased IKs in LQT2 hiPSC-CMs could be part of a compensatory mechanism to assist the CMs in repolarization. The aim of our further studies is to investigate the presence of various compensatory repolarization mechanisms in LQT2 asymptomatic hiPSC-CMs and if the increase in ICaL is a specific phenomenon for symptomatic hiPSC-CMs, as implicated by Chai et al. [14].

### 4.3. Limitations of the Study

The limitations of our study include not using control hiPSC-CMs derived from non-carriers in the same family. hiPSC-CM lines from the CRISPR/Cas9 corrected controls and corrected mutations were not applicable here, since the mutation was the same in the two LQT2 lines analyzed but the clinical phenotype was different. Electrophysiological data from measurement of IKs and ICaL, studying the connexin 43 expression and detailed genetic studies to identify gene variants were not done for this study. These experiments could help understand differences between asymptomatic and symptomatic mutation carriers further. Another improvement of this study could be achieved by having more than just one familial pair of asymptomatic and symptomatic mutation carriers each from both sexes, to be certain of the disease-specific effect rather than gender-specific effects which may play a role. We also acknowledge the variable phenotype of hiPSC-CMs in general and urge to exercise caution while reading the results from a clinical perspective.

## 5. Conclusions

Clinically, the severity of disease presentation and variable penetrance is a cause of concern. We found differences at CM-aggregate level in the phenotype in-vitro between hiPSC-CMs from LQT2 asymptomatic and symptomatic individuals with hiPSC-CMs aggregates from symptomatic patient being more arrhythmic on IKr and IKs block. Furthermore, characteristic arrhythmia of LQT2 on β-adrenergic stimulation was observed at hiPSC-CM aggregate level in LQT2 asymptomatic and symptomatic hiPSC-CMs, with increased arrhythmia observed in the symptomatic. Taken together, our findings demonstrated that the hiPSC-CMs can reveal the different phenotypes of mutation carriers having a different clinical phenotype. The reason for this different behavior clinically and at cellular level is still under investigation.

## Supplementary Materials

The following are available online at https://www.mdpi.com/2073-4409/9/5/1153/s1, Figure S1: Representative hiPSC colony pictures; Figure S2: Endo and Exp PCR in the characterization of hiPSC lines; Figure S3: Embryoid body (EB) assay for the detection of ectoderm, mesoderm and endoderm, Figure S4: Normal karyotype found in all the hiPSC lines, Figure S5: Pluripotency marker expression on hiPSC under immunofluorescent staining, Figure S6: Representative Ca^2+^ transients at baseline and under adrenaline, Figure S7: Adrenaline increases the beat rate and reduces the field potential duration, Figure S8: Allelic imbalance assay, Figure S9: ECG from healthy individual, asymptomatic and symptomatic LQT2 mutation carriers.
