# Supplementary material for: hiPSC-Derived Cardiomyocyte Model of LQT2 Syndrome Derived from Asymptomatic and Symptomatic Mutation Carriers Reproduces Clinical Differences in Aggregates but Not in Single Cells
Source: Cells. 2020 May 7;9(5):1153. doi: 10.3390/cells9051153 (PMC7290503; doi:10.3390/cells9051153)
Supplement: Supplementary file 1 [file cells-09-01153-s001.pdf]

Supplementary information for:

hiPSC-derived cardiomyocyte model of LQT2 syndrome derived from asymptomatic and symptomatic mutation carriers reproduces clinical differences in aggregates but not in single cells

Disheet Shah[1*], Chandra Prajapati[1], Kirsi Penttinen[1], Reeja Maria Cherian[1], Jussi T. Koivumäki[1], Anna Alexanova[1], Jari Hyttinen[1], Katriina Aalto-Setälä[1,2]

Affiliation 1; Faculty of Medicine and Health Technology and BioMediTech Institute, Tampere University, Tampere, Finland

Affiliation 2; Heart Hospital, Tampere University Hospital, Tampere, Finland

Characterization of lines-

Full characterization of Control1 including EB assay was carried out in a previous manuscript from the same group [1, 2], characterization of Control (UTA.04511.WT) and LQT2 (UTA.03412.LQT2A, UTA.03417.LQT2A, UTA.03809.LQT2A, UTA.03810.LQT2A) hiPSC lines is presented here.

Figure S1: Representative hiPSC colony pictures

UTA.04511.WT p24 (colony also used for immunostaining)

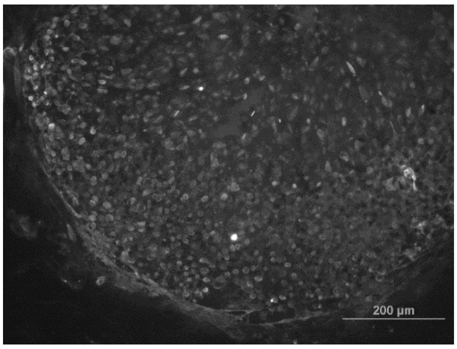

UTA.03412.LQT2_P34
(colony also used for immunostaining)

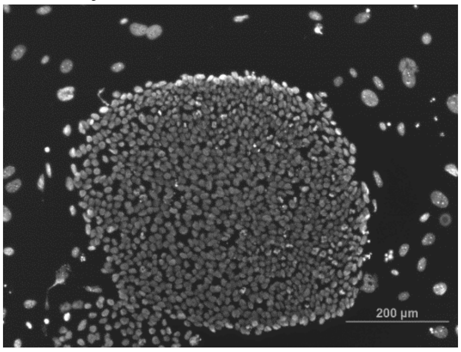

UTA.03417.LQT2
(colony also used for immunostaining)

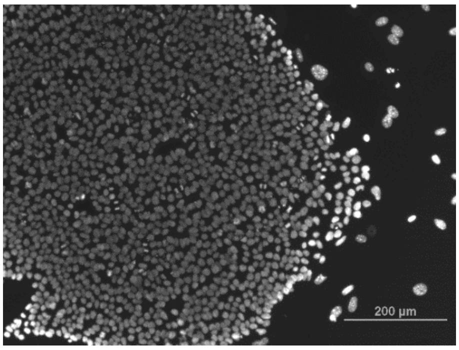

UTA.03809.LQT2_P25

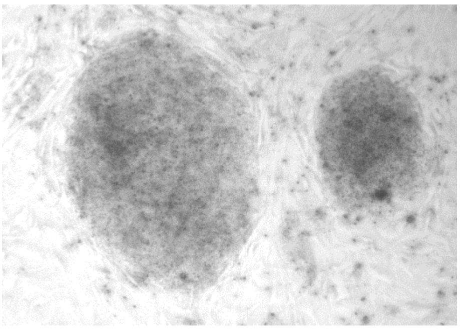

UTA.03810.LQT2_P27

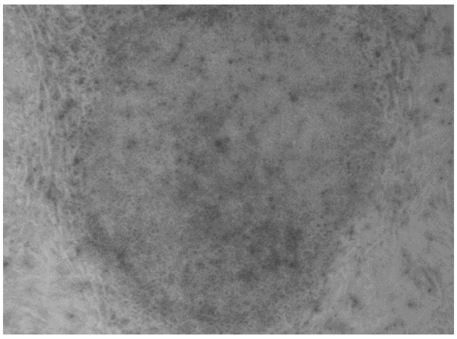

Figure S1: Representative hiPSC colony pictures; Control (UTA.04511.WT) and LQT2 (UTA.03412.LQT2A, UTA.03417.LQT2A, UTA.03809.LQT2A, UTA.03810.LQT2A) hiPSC lines under phase contrast microscopy.

Figure S2: Endo and Exo PCR in characterization of hiPSC lines

endoPCR

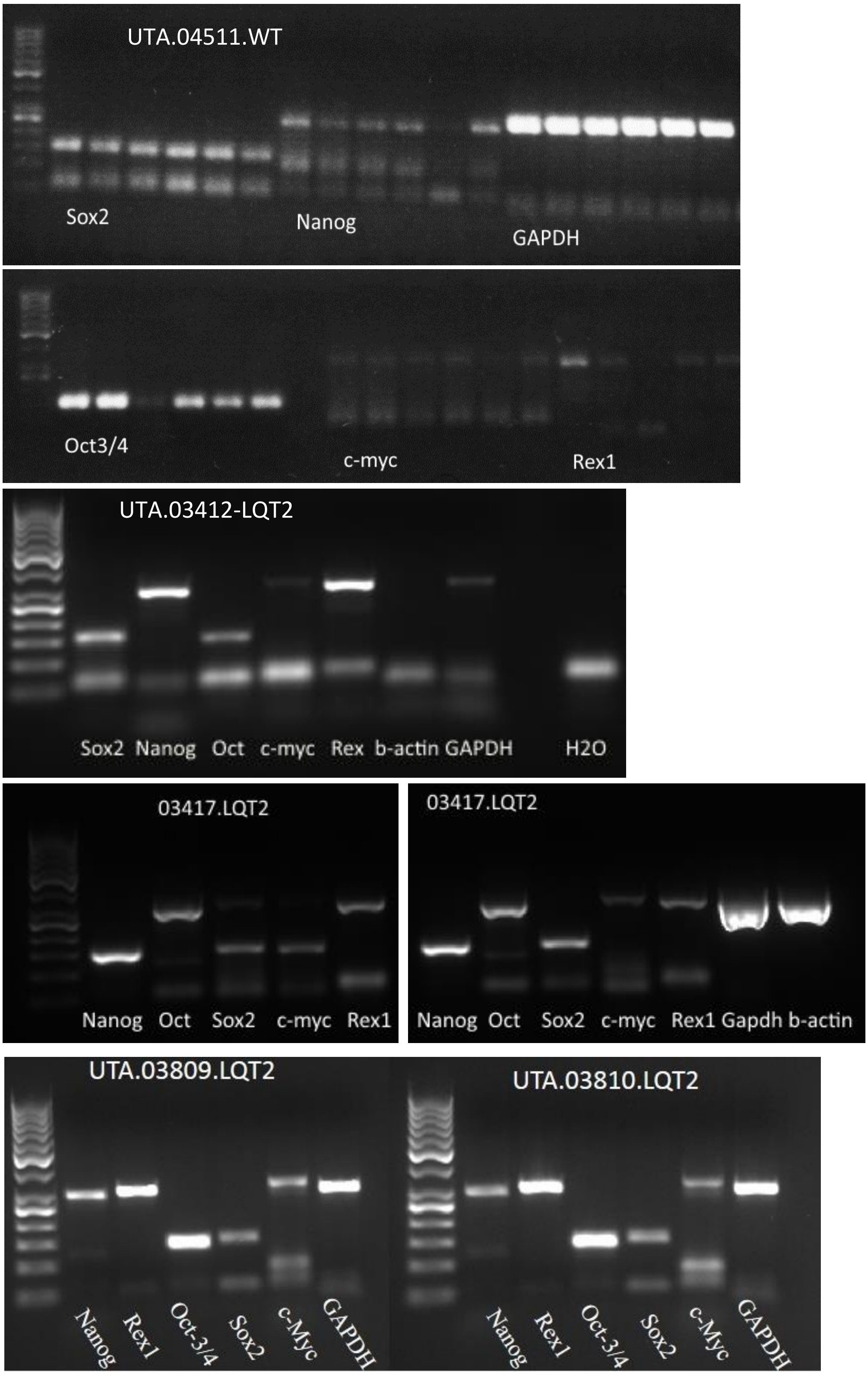

exoPCR

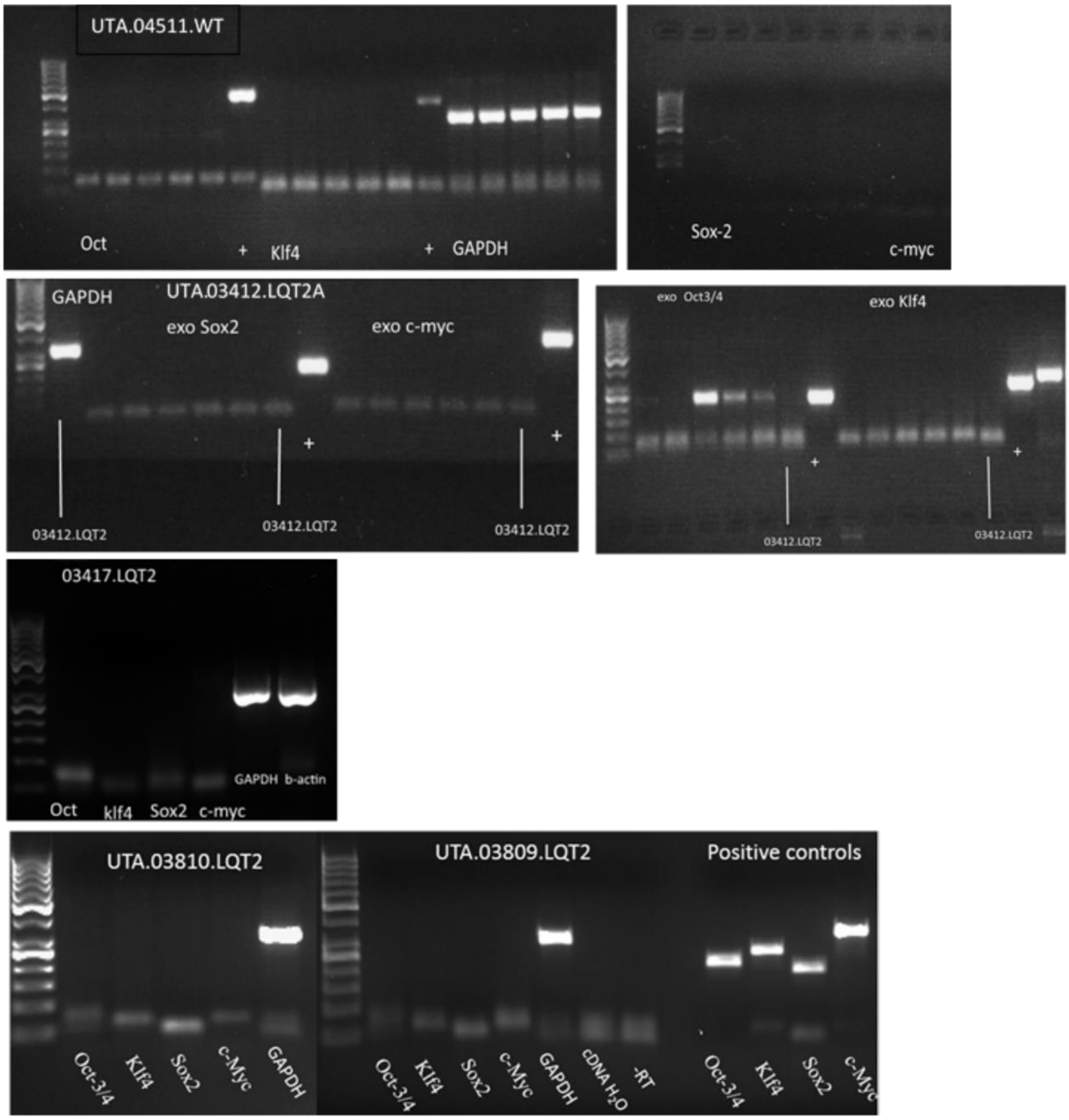

Figure S2: Expression of endogens in Control (UTA.04511.WT) and LQT2 (UTA.03412.LQT2A, UTA.03417.LQT2A, UTA.03809.LQT2A, UTA.03810.LQT2A) hiPSC lines; *REX1* (306 bp), *SOX2* (151 bp), *Nanog* (287 bp), and *c-MYC* (328 bp). *GAPDH* (302 bp) was used as a housekeeping control transcript. And the silenced virally transfected Sendai exogenes, *exo-OCT4* (483 bp), *exo-c-MYC* (532 bp), exo-*SOX2* (451 bp) and *exo-KLF4* (410 bp), in the hiPSCs 1 week after transfection GAPDH was used as a control.

Figure S3: Embryoid body (EB) assay for detection of ectoderm, mesoderm and endoderm

EB PCR

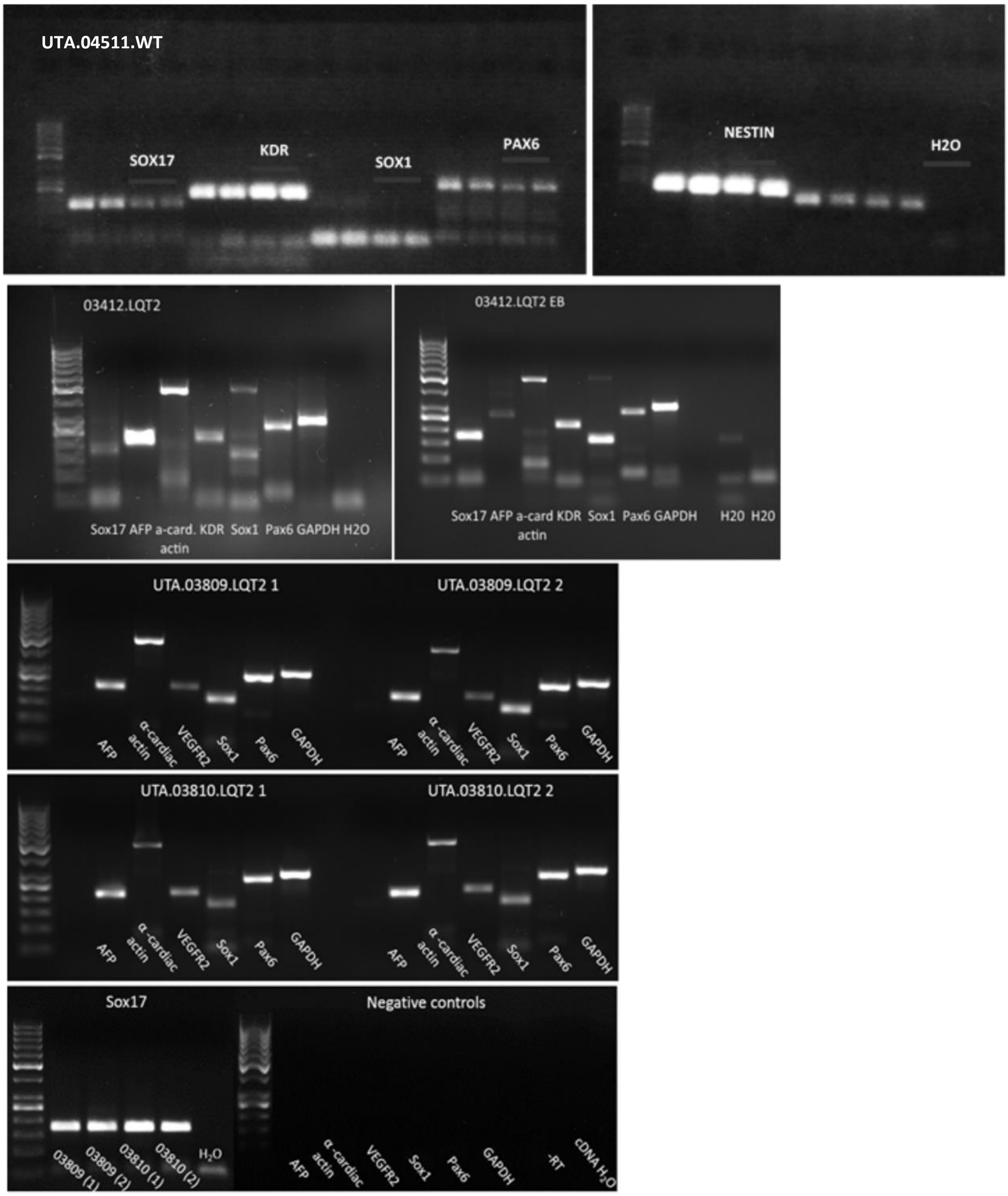

Figure S3. Expression of markers for endoderm, mesoderm and ectoderm in Control (UTA.04511.WT) and LQT2 (UTA.03412.LQT2A, UTA.03417.LQT2A, UTA.03809.LQT2A, UTA.03810.LQT2A) hiPSC lines.

Figure S4: Normal karyotype found in all the hiPSC lines

UTA.04511.WT P30

Normalized Ratios

Probe	/F	/M	Chromosomal Location
01C	0.98	1.00	
02C	1.00	0.99	
03C	0.98	1.02	
04C	1.00	1.03	
05C	1.07	1.04	
06C	1.02	1.04	
07C	1.06	1.04	
08C	0.97	0.96	
09C	0.97	1.01	
10C	1.02	1.00	
11C	0.93	0.97	
12C	1.02	1.01	
13C	1.02	0.99	
14C	0.96	0.99	
15C	0.99	0.99	
16C	1.02	1.03	
17C	0.98	0.98	
18C	0.98	0.98	
19C	1.01	1.03	
20C	1.03	0.97	
21C	1.03	0.99	
22C	1.00	1.05	
Auto	1.03	0.99	
X1;ptel	0.70	0.99	-
X2;pcen	0.64	0.97	-
X3;qcen	0.77	0.97	-
X4;qtel	0.71	0.93	-
Y1;ptel	1.48	0.96	-
Y2;pcen	2.50	0.99	-
Y3;qcen	1.35	1.05	-
Y4;qtel	2.18	0.99	-

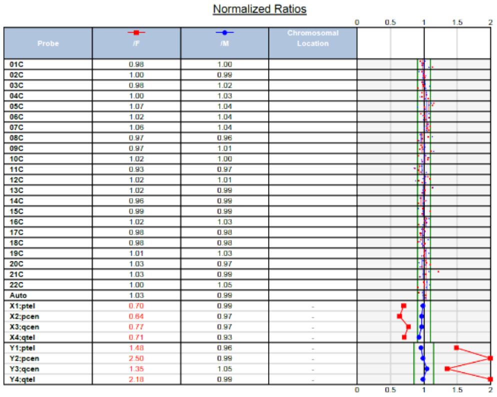

UTA.03412.LQT2 P29

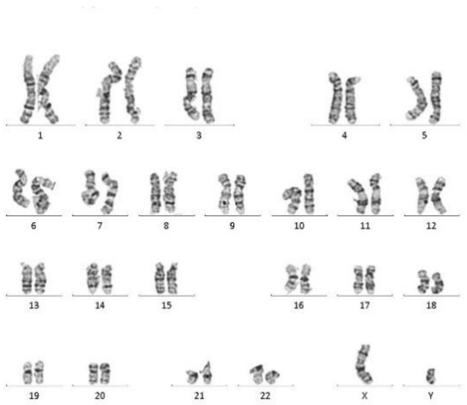

UTA.03417.LQT2 P50

Normalized Ratios

Probe	/F	/M	Chromosomal Location
01C	1.00	0.97	
02C	0.94	0.97	
03C	1.01	0.99	
04C	0.95	0.99	
05C	0.97	0.99	
06C	0.98	1.00	
07C	1.03	1.00	
08C	0.97	1.00	
09C	1.00	1.00	
10C	1.00	1.01	
11C	1.05	1.03	
12C	1.03	1.02	
13C	0.96	0.97	
14C	1.07	1.05	
15C	1.00	1.03	
16C	0.96	0.96	
17C	1.01	1.03	
18C	1.00	0.98	
19C	0.99	0.99	
20C	0.99	1.01	
21C	1.01	1.04	
22C	1.00	1.01	
Auto	1.00	1.02	
X1;ptel	0.71	1.01	-
X2;pcen	0.70	0.98	-
X3;qcen	0.76	0.97	-
X4;qtel	0.75	0.99	-
Y1;ptel	1.31	1.01	-
Y2;pcen	2.23	0.97	-
Y3;qcen	1.27	1.02	-
Y4;qtel	1.67	0.97	-

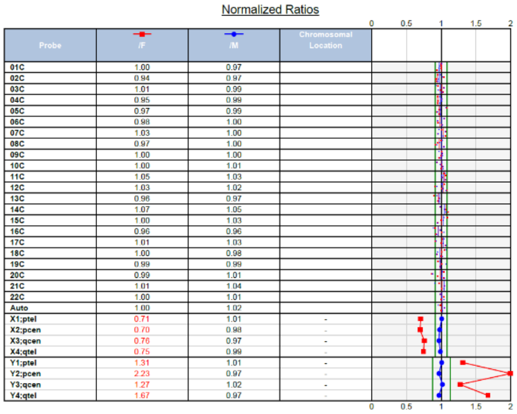

UTA.03810.LQT2 P22

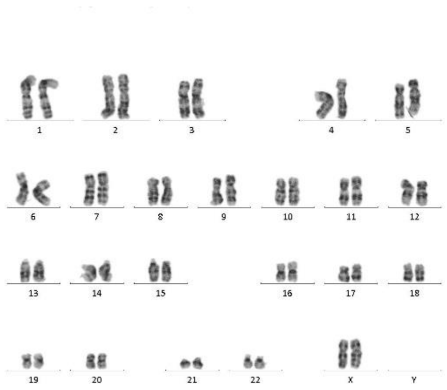

UTA.03809.LQT2 P10

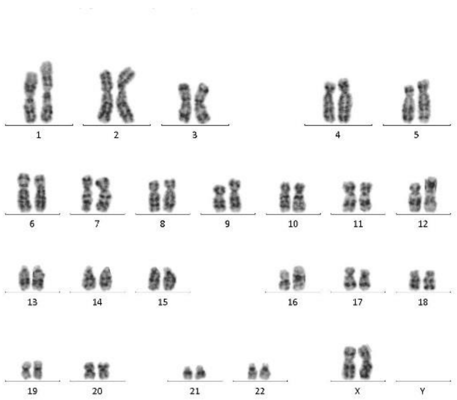

Figure S4. Karyotype analysis of hiPSC colonies from Control (UTA.04511.WT) and LQT2 (UTA.03412.LQT2A, UTA.03417.LQT2A, UTA.03809.LQT2A, UTA.03810.LQT2A) hiPSC lines did not show any major karyotype aberrations.

Figure S5: Pluripotency marker expression on hiPSC under immunofluorescent staining

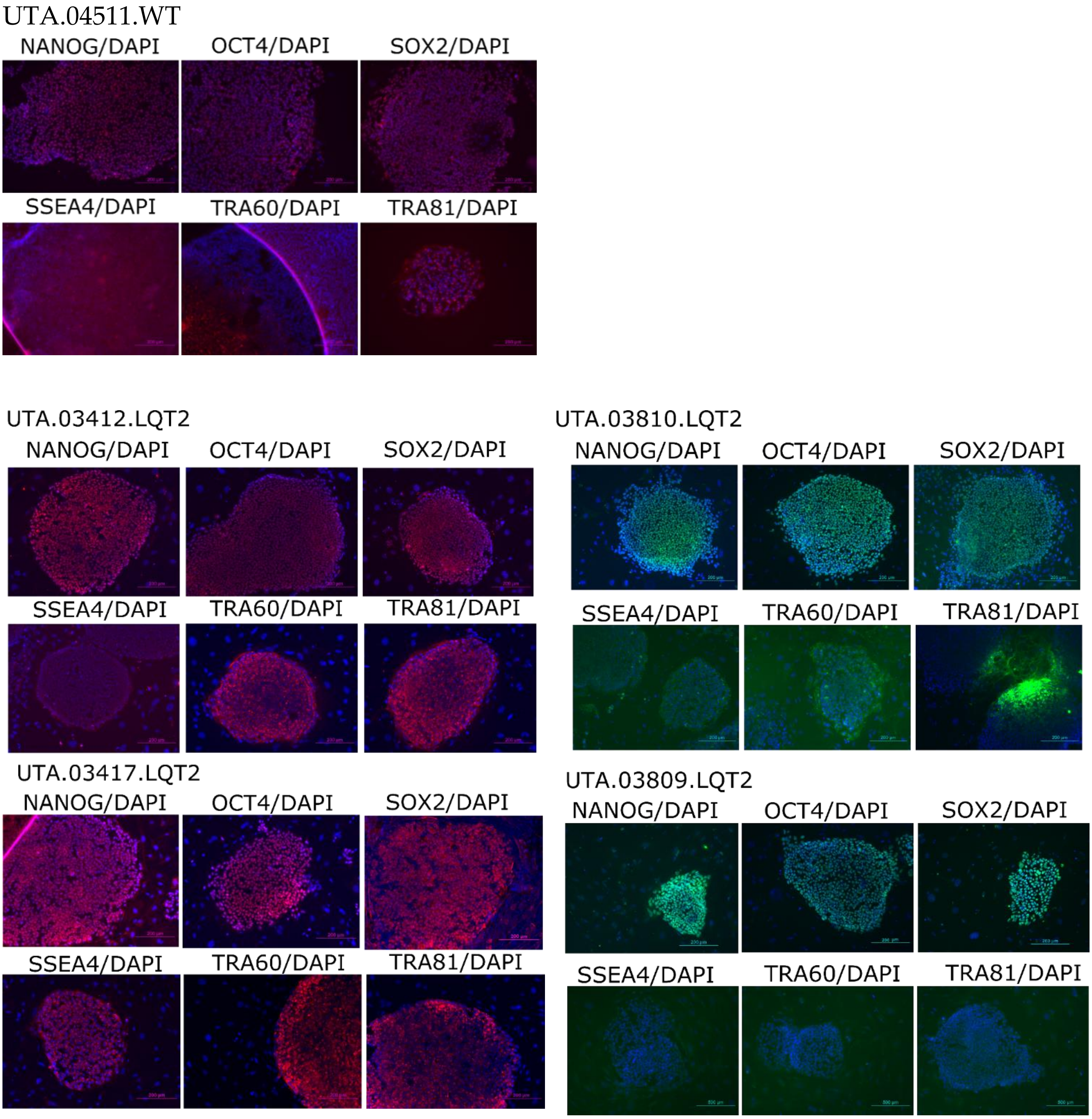

Figure S5. Representative immunofluorescence staining from hiPSC colonies from Control (UTA.04511.WT) and LQT2 (UTA.03412.LQT2A, UTA.03417.LQT2A, UTA.03809.LQT2A, UTA.03810.LQT2A expressing NANOG, OCT4, SOX2, SSEA4, TRA-1-60, and TRA-1-81. Stainings were essentially similar in all the lines.

Figure S6: Representative Ca2+ transients at baseline and under adrenaline

A)

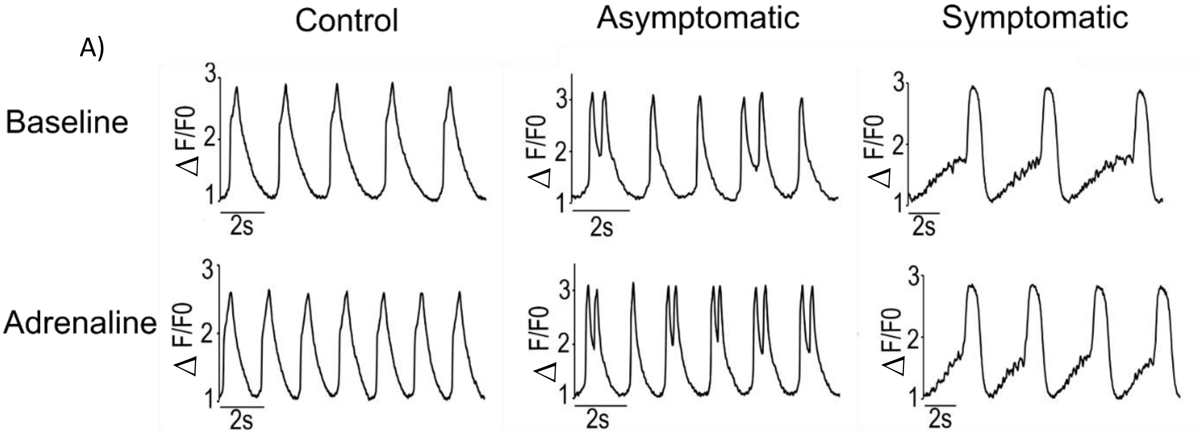

Figure S6: Representative calcium transients from control, asymptomatic and symptomatic hiPSC-CMs at baseline (top trace) and at 10nM adrenaline (bottom).

B)

CaT arrhythmias defined with more clarity -

- Oscillation, if there were two or more peaks in the same event without reaching the baseline, more precisely the lowest point between these peaks was 10-90% of the amplitude of a normal peak in the trace.
- Varying amplitude (VA) if the amplitude of the peaks were varying continuously in the trace and the amplitude of the varying peak was 10–90% of the amplitude of a normal peak in the trace.
- Plateau abnormality (PA) if the decay time of the Ca2+ was prolonged by displaying a notch in the decay and therefore causing the peak duration to increase at least 60% from a normal peak in the trace
- Rise delay (RD) if the rise time of the Ca2+ was prolonged by displaying a notch in the rise and therefore causing the peak duration to increase at least 60% from a normal peak in the trace.

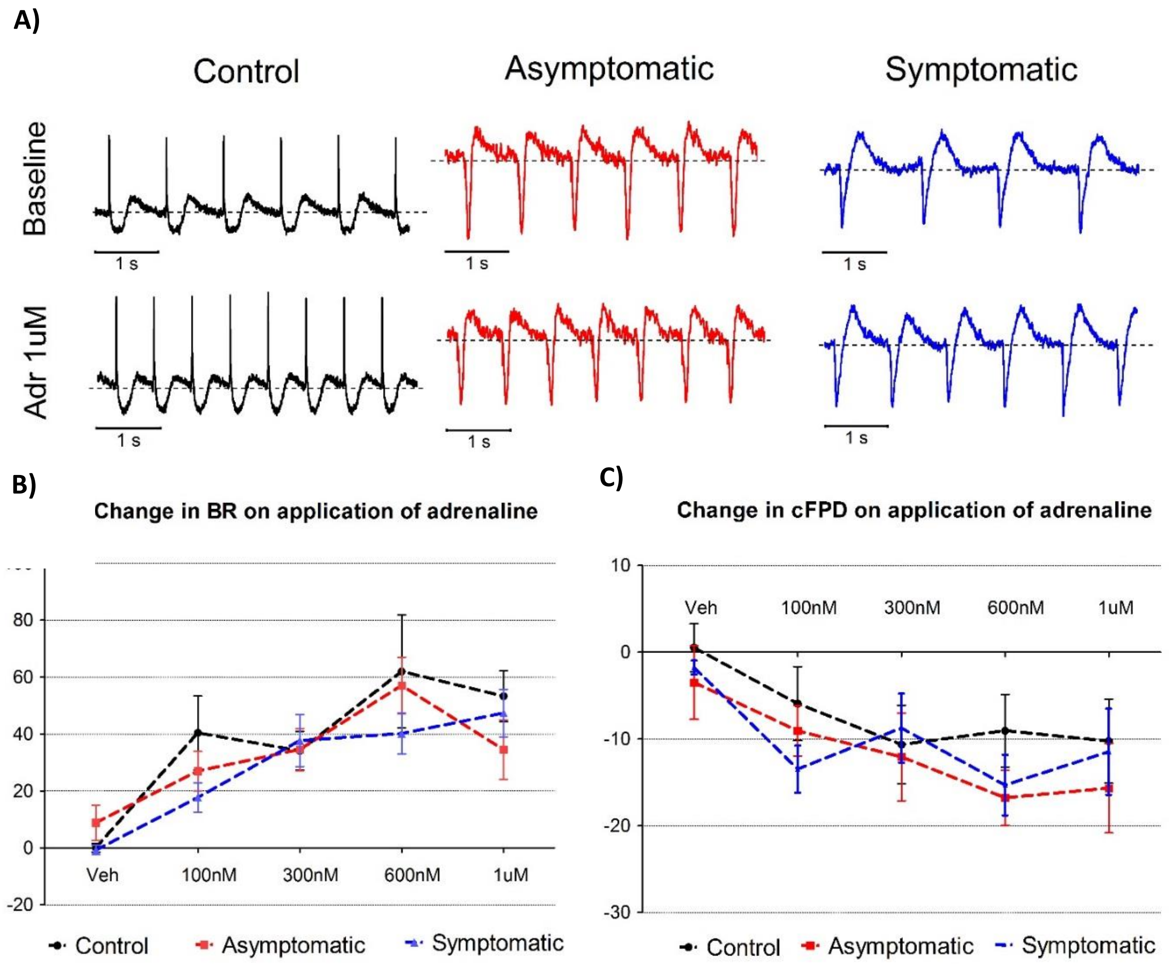

Figure S7: Adrenaline increases the beat rate and reduces the field potential duration

A) The plots show representative field potential traces from control, asymptomatic and symptomatic hiPSC-CM aggregates at baseline (top trace) and at 1 µM adrenaline (bottom). B-C) show responses of control, asymptomatic and symptomatic hiPSC-CM aggregates to percentage change in the beat rate (BR) and corrected filed potential duration (cFPD) on application of vehicle distilled H_2O (veh) and 100 nM, 300 nM, 600 nM and 1 µM adrenaline; the n numbers control, asymptomatic and symptomatic hiPSC-CM aggregates were; vehicle n=22, 10, 20; 100nM n= 25, 12, 22; 300nM n= 24, 13, 21; 600nM n= 22, 14, 20; 1 µM n=25, 14, 22 respectively. Data is presented as mean ± s.e.m.

Figure 8: Allelic imbalance assay

<u>Generation of plasmid standards for allelic imbalance determination</u>

WT plasmid was generated from cDNA sample of UTA.04602.WT cell line by amplifying genetic site of *HERG* with forward and reverse primers (Supplementary Table 1) containing restriction sites for BamHI (Thermo Fisher Scientific Inc., Waltham, MA, USA) and NotI (Thermo Fisher Scientific Inc.) enzymes, respectively. The wild type allele fragment (320 bp)

was inserted into pBluescript SK+ plasmid (Addgene, Cambridge, MA, USA) via restriction sites for BamHI and NotI.

Mutant plasmid of *HERG*-FinA was generated from its WT plasmid by using QuikChange II Site-Directed Mutagenesis Kit (Agilent Technologies Inc., Santa Clara, CA, USA). Mutagenic primers are listed in Supplementary Table 1. Both plasmid constructs were sequenced to verify the presence of correct allele fragment.

Supplementary Table 1. Primers used in allelic imbalance assay

Assay	Primer/probe	Sequence
WT plasmid generation	Forward primer with BamHI restriction site	GCGGGATCCCTGAAGACTGCG CGGCTGCT
	Reverse primer with NotI restriction site	GCGGCGGCCGCGGCCGACACG TTGCCGAAGA
HERG-FinA plasmid generation	Mutagenesis sense primer	GCGGCCGTGCTGTTCTCGCTCA TGTGCA
	Mutagenesis antisense primer	TGCACATGAGCGAGAACAGCA CGGCCGC
TaqMan SNP Genotyping assay for allelic imbalance determination	Forward primer	AGCTGGATCGCTACTCAGAGT
	Reverse primer	GCGATGAGCGCAAAGGT
	Probe wild type VIC	CACATGAGCAAGAACA
	Probe *HERG*-FinA FAM	CACATGAGCGAGAACA

RT-qPCR Data analysis

The data acquired from post-read was evaluated with 7300 SDS software (v1.4) provided with the Applied Biosystems 7300 Real-Time PCR system. The software identified dye components and determined the contribution of each dye in the raw data. Log2 wild type/mutant plasmid ratios, [log2(WT/MUT)], were plotted versus corresponding average ΔCt (Ct mut - Ct wt) values of the triplicates. The standard curve was fit between the generated points using least squares fit. The equation of the standard curve was used to calculate [log2 (WT/MUT)] values for the symptomatic and asymptomatic hiPSC-CMs from the mean ΔCt values. The calculated values were then plotted in the same graph with the standard curve to assess the expression ratios of the two *HERG* alleles by the location of the generated points. Error bars represent s.e.m.

Figure 9: ECG from healthy individual, asymptomatic and symptomatic LQT2 mutation carriers.

A.

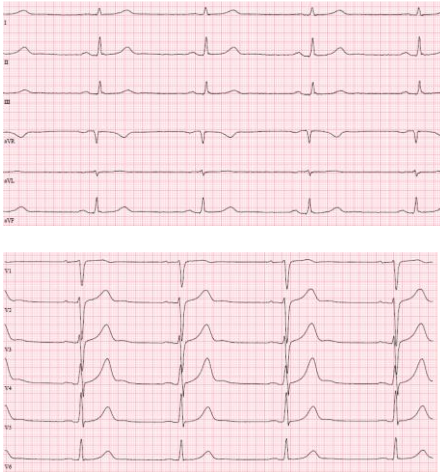

B.

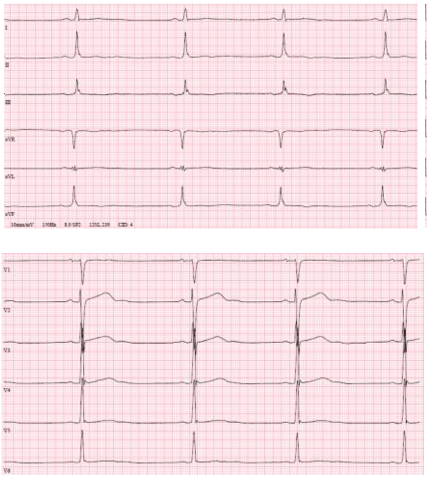

C.

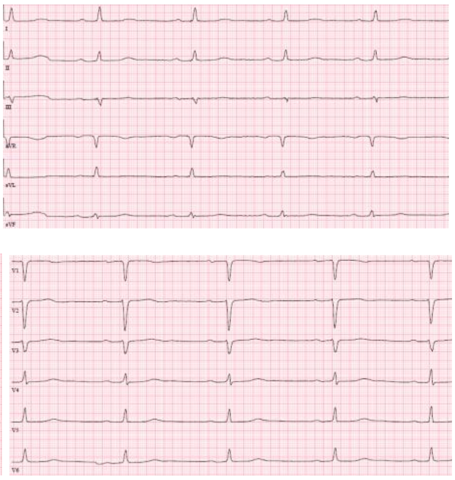

D.

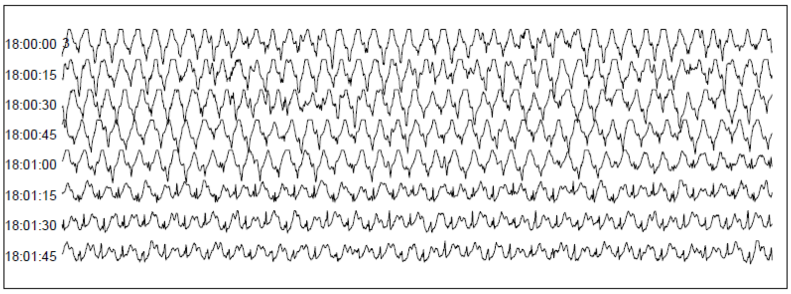

Figure S9: ECG from a healthy individual (A), the asymptomatic mutation carrier (B) and from the symptomatic mutation carrier (C). The QT/QTC in the control ECG is 456/415 ms, in asymptomatic 450/398 ms and in symptomatic 492/470 ms. (D) The presence of NSVT in the symptomatic individual during 24 h Holter recording.
